# Patient-specific iPSC-derived cardiomyocytes reveal aberrant activation of Wnt/β-catenin signaling in *SCN5A*-related Brugada syndrome

**DOI:** 10.1186/s13287-023-03477-3

**Published:** 2023-09-08

**Authors:** Dongsheng Cai, Xiaochen Wang, Yaxun Sun, Hangping Fan, Jingjun Zhou, Zongkuai Yang, Hangyuan Qiu, Jue Wang, Jun Su, Tingyu Gong, Chenyang Jiang, Ping Liang

**Affiliations:** 1 Department of Cardiology, Sir Run Run Shaw Hospital, Zhejiang University School of Medicine, 3 Qingchun East Road, Hangzhou, 310016 China; 2 Key Laboratory of Combined Multi-Organ Transplantation, Ministry of Public Health, the First Affiliated Hospital, Zhejiang University School of Medicine, 79 Qingchun Road, Hangzhou, 310003 China; 3 Institute of Translational Medicine, Zhejiang University, Hangzhou, 310029 China

**Keywords:** iPSC-CMs, Brugada syndrome, *SCN5A*, Na_v_1.5, Wnt/β-catenin signaling

## Abstract

**Background:**

Mutations in the cardiac sodium channel gene *SCN5A* cause Brugada syndrome (BrS), an arrhythmic disorder that is a leading cause of sudden death and lacks effective treatment. An association between *SCN5A* and Wnt/β-catenin signaling has been recently established. However, the role of Wnt/β-catenin signaling in BrS and underlying mechanisms remains unknown.

**Methods:**

Three healthy control subjects and one BrS patient carrying a novel frameshift mutation (T1788fs) in the *SCN5A* gene were recruited in this study. Control and BrS patient-specific induced pluripotent stem cells (iPSCs) were generated from skin fibroblasts using nonintegrated Sendai virus. All iPSCs were differentiated into cardiomyocytes using monolayer-based differentiation protocol. Action potentials and sodium currents were recorded from control and BrS iPSC-derived cardiomyocytes (iPSC-CMs) by single-cell patch clamp.

**Results:**

BrS iPSC-CMs exhibited increased burden of arrhythmias and abnormal action potential profile featured by slower depolarization, decreased action potential amplitude, and increased beating interval variation. Moreover, BrS iPSC-CMs showed cardiac sodium channel (Na_v_1.5) loss-of-function as compared to control iPSC-CMs. Interestingly, the electrophysiological abnormalities and Na_v_1.5 loss-of-function observed in BrS iPSC-CMs were accompanied by aberrant activation of Wnt/β-catenin signaling. Notably, inhibition of Wnt/β-catenin significantly rescued Na_v_1.5 defects and arrhythmic phenotype in BrS iPSC-CMs. Mechanistically, *SCN5A*-encoded Na_v_1.5 interacts with β-catenin, and reduced expression of Na_v_1.5 leads to re-localization of β-catenin in BrS iPSC-CMs, which aberrantly activates Wnt/β-catenin signaling to suppress *SCN5A* transcription.

**Conclusions:**

Our findings suggest that aberrant activation of Wnt/β-catenin signaling contributes to the pathogenesis of *SCN5A*-related BrS and point to Wnt/β-catenin as a potential therapeutic target.

**Supplementary Information:**

The online version contains supplementary material available at 10.1186/s13287-023-03477-3.

## Background

Brugada syndrome (BrS) was first reported in 1992 and is a primary electrical disorder characterized by ST-segment elevation in the right precordial electrocardiogram (ECG) leads [1]. Patients with BrS have an increased risk of sudden cardiac death (SCD) due to ventricular fibrillation (VF) [2]. The estimated prevalence of BrS ranged from 1 in 5000 to 1 in 200 around the world, with a much higher prevalence in males [3]. It is worth emphasizing that BrS is a leading cause of sudden death in men under 50 years old, particularly in Southeast Asia [4]. Unfortunately, there are few treatments for BrS at the moment [5]. Implantable cardioverter-defibrillation (ICD) is the only proven effective treatment for the prevention of SCD in BrS patients [2, 6]. However, such therapy is invasive and associated with various complications [3]. Therefore, it is of great clinical significance to find more effective treatment options for BrS patients.

Genetic studies have demonstrated that BrS is related to mutations in several genes encoding different subunits of sodium, L-type calcium, and potassium channels [7]. The most commonly disease-related mutations have been found in *SCN5A*, which encodes the α-subunit of the voltage-gated cardiac sodium channel (Na_v_1.5) and has been identified in 20–25% of BrS patients [8, 9]. Mutations in the *SCN5A* gene result in a reduction in the cardiac sodium current, which is believed to play a key role in the pathogenesis of *SCN5A*-related BrS [10].

Wnt/β-catenin signaling, also known as the canonical Wnt pathway, plays critical roles in numerous physiological and pathological progress, including embryonic development, apoptosis and adult tissue homeostasis [11]. The association between Na_v_1.5 and Wnt/β-catenin signaling has been recently established [12, 13]. Activation of Wnt/β-catenin signaling can lead to the suppression of *SCN5A* transcription and cardiac sodium current density [14]. Notably, inhibition of Wnt/β-catenin significantly increases cardiac sodium current in HL-1 cardiomyocytes [15]. In addition, aberrant activation of Wnt/β-catenin signaling is discovered in various heart diseases such as myocardial infarction, heart failure, cardiac hypertrophy and inherited cardiac diseases [16–20]. However, the role of Wnt/β-catenin signaling in the pathogenesis of *SCN5A*-related BrS and underlying mechanisms remains unknown.

Human-induced pluripotent stem cell-derived cardiomyocytes (iPSC-CMs) have been widely used for studying cardiac arrhythmic disorders [21–23], thus providing a promising opportunity for recapitulating disease phenotypes in a patient-specific manner. To date, almost all iPSC-based studies for investigating *SCN5A*-related BrS have stayed on exploring the cellular phenotypes [24–30]. The molecular mechanisms of arrhythmogenesis in *SCN5A*-related BrS are still largely unknown.

Here, we demonstrated the use of patient-specific iPSC-CMs carrying the *SCN5A* mutation affected by BrS as a cellular model to elucidate potential mechanisms underlying the alteration of Wnt/β-catenin signaling in the pathogenesis of this disease.

## Methods

### Culture and maintenance of skin fibroblasts

Freshly isolated skin biopsies were rinsed with Dulbecco’s Phosphate-Buffered Saline (DPBS) (Gibco, C14190500BT) and transferred into a 1.5-ml tube. Tissue was minced in collagenase I (1 mg/mL in Dulbecco’s modified Eagle medium (DMEM), Gibco, C11995500BT) and allowed to digest for 6 h at 37 °C. Dissociated skin fibroblasts were plated and maintained with DMEM containing 10% FBS (Gibco, 10091148), 100 U/mL Penicillin and 100 μg/mL Streptomycin (Gibco, 15140122) at 37 °C, 95% air and 5% CO_2_ in a humidified incubator. All skin fibroblasts were used for reprogramming within 5 passages.

### Generation of iPSCs

Somatic reprogramming was used to generate control and BrS iPSC lines from skin fibroblasts using CytoTune-iPS 2.0 Sendai Reprogramming Kit following the manufacturer’s instructions (Invitrogen, A16517).

### Culture and maintenance of iPSCs

The iPSCs were cultured in feeder-free mTeSR1 (STEMCELL Technologies, 85850) media on matrigel-coated (Corning, 354277) plates at 37 °C with 5% (vol/vol) CO_2_. The media were daily changed, and iPSCs were passaged every 3–4 days using Accutase (STEMCELL Technologies, 07920).

### Karyotyping

Chromosome analysis by G-banding was achieved using iPSCs at passage 20 by the Prenatal Diagnosis Center of Hangzhou Women's Hospital. At least 20 metaphase cells were analyzed at 300–400 band level.

### Alkaline phosphatase staining

Alkaline phosphatase (ALP) staining was performed using the VECTOR Blue Alkaline Phosphatase Substrate Kit (Vector Laboratories, SK-5300) following the manufacturer’s instructions.

### Cardiac differentiation

The iPSC-CMs were generated using a 2D monolayer differentiation protocol. Briefly, ~ 10^5^ undifferentiated iPSCs were dissociated and replated into matrigel-coated 6-well plates. The iPSCs were cultured and expanded to 85% confluence and then, treated for 2 days with 6 μM CHIR99021 (Axon Medchem, 1386) in RPMI 1640 (Gibco, C11875500BT) with B27 supplement minus insulin (Gibco, A1895601) (RPMI + B27-Insulin) to activate Wnt signaling pathway. On day 2, cells were placed in RPMI + B27-Insulin with CHIR99021 removal. On days 3–4, cells were treated with 5 μM IWR-1 (Sigma-Aldrich, 681669) to inhibit Wnt signaling pathway. On days 5–6, cells were removed from IWR-1 treatment and placed in RPMI + B27-Insulin. From day 7 onwards, cells were placed and cultured in RPMI 1640 and B27 supplement with insulin (Gibco, 17504044) (RPMI + B27 + Insulin) until beating was observed. Cells were glucose-starved for 3 days with RPMI + B27 + Insulin for the purification. Cardiomyocytes of day 30–40 after cardiac differentiation were utilized for downstream functional assays.

### Immunofluorescent staining

Cells were fixed with 4% paraformaldehyde (PFA) for 15 min, permeabilized with 0.1% Triton X-100 (Sangon Biotech, A110694) for 5 min, and blocked with 3% bovine serum albumin (Sigma-Aldrich, A1933) for 1 h. Cells were subsequently stained with appropriate primary antibodies and AlexaFluor conjugated secondary antibodies. Primary antibodies include NANOG (Santa Cruz Biotechnology, sc-33759, 1:200), SOX2 (Abcam, ab97959, 1 μg/ml), OCT4 (Santa Cruz Biotechnology, sc-8628, 1:500), SSEA-4 (Abcam, ab16287, 1:500) TNNT2 (Abcam, ab45932, 1:400), α-actinin (Abcam, ab137346, 1:500), Na_v_1.5 (Alomone labs, ASC-005, 1:200) and β-catenin (Abcam 237983, 1ug/ml). Secondary antibodies include AlexaFluor® 647 (Abcam, ab150079, 1:500), AlexaFluor® 594 (Abcam, ab150108, 1:500) and AlexaFluor® 488 (Abcam, ab150113, 1:500; Invitrogen, A11008, 1:500). Nuclei were stained with DAPI (Roche Diagnostics, 1023276001, 1 μg/ml). Pictures were taken with 60 × objective on confocal microscope (Nikon, A1) using NIS-Elements AR software (Nikon).

### TOPflash assay

Wnt signaling activity was evaluated using the well-described TOPflash assay [31]. Briefly, Wnt-specific TOPflash luciferase reporter and Renilla luciferase plasmid were co-transfected into control and BrS iPSC-CMs. Cells were harvested and assayed 24 h after transfection using the Dual-Luciferase Reporter Assay System (Promega). The TOPflash activity was normalized to Renilla luciferase signals.

### Real-time quantitative PCR (qPCR)

The iPSC-CMs were lysed using Trizol (Invitrogen) followed by RNA extraction. RNA concentration was measured using UV spectrophotometry at 260 nm (Nanodrop 2000, Thermo Scientific). cDNA was obtained using the High Capacity cDNA Reverse transcription Kit (Applied Biosystems). qPCR was performed using SYBR Green PCR Master Mix (Takara). Primer sequences used in this study are listed in Additional file 1: Table S1. Each reaction was run in triplicates using an Applied Biosystems Viia7 Dx (Thermo Fisher Scientific). Gene expression values were normalized to the average expression of housekeeping gene *GAPDH*.

### Western blot

The iPSC-CMs were detached with TrypLE and then, pelleted at 1000 rpm for 5 min at 4 °C. After washing with PBS, the pellets were re-suspended in 50–100 μl lysis buffer. Lysates were placed on ice for 30 min, and then, the supernatants were collected after centrifuging at 12,000 rpm for 15 min. Cytosolic and nuclear fractions were extracted with NE-PER™ Nuclear and Cytoplasmic Extraction Reagents kit (Thermo Scientific, 78,833) according to the manufacturer’s instruction. Protein concentration was measured using a BCA kit (Pierce, 23227). Western blot was performed using standard protocol with the following antibodies: Na_v_1.5 (Alomone Labs, ASC-005, 1:500), total β-catenin (Abcam, ab32572, 1:5000), active β-catenin (Abcam, ab246504, 1:1000), Lamin B1 (Abcam, ab16048, 1:5000) and GAPDH (Abmart, M200006, 1:5000). Intensity values for each band were determined as the integrated density (sum of pixel values) within a fixed area using Quantity One software (Biorad).

### Co-immunoprecipitation (Co-IP)

This assay was performed using Pierce™ Classic Magnetic IP/Co-IP Kit (Thermo Fisher Scientific). Briefly, iPSC-CMs were lysed in 1 × lysis/wash buffer with protease inhibitor on ice for 5 min. The lysate was then centrifuged at 12,000 rpm for 10 min at 4 °C. The centrifuged lysate was diluted to 500 μl and incubated with the anti-β-catenin or anti-Na_v_1.5 antibodies for 2 h at room temperature. Next, the antigen sample/antibody mixture was incubated with 25 μl pre-washed magnetic beads for 1 h with mixing at room temperature. Bead-bound proteins were dissociated in the elution buffer for 10 min. The associated protein was finally identified by Western blots.

### Patch clamp recordings

The iPSC-CMs were mechanically and enzymatically dissociated to obtain single cells, which were seeded on matrigel-coated glass coverslips (Warner Instruments). Cells with spontaneous beatings were selected, and action potentials were recorded using an EPC-10 patch clamp amplifier (HEKA). Continuous extracellular solution perfusion was achieved using a rapid solution exchanger (Bio-logic Science Instruments, RSC-200). All signals were acquired using PatchMaster software (HEKA) and filtered at 1 kHz and digitized at 10 kHz. Data analyses were performed using Igor Pro (Wavemetrics) and GraphPad Prism (GraphPad Software). A TC-344C dual channel heating system (Warner Instruments) was used to maintain the temperature at 35.5–37 °C. Tyrode’s solution was used as the external solution containing 140 mM NaCl, 5.4 mM KCl, 1 mM MgCl_2_, 10 mM glucose, 1.8 mM CaCl_2_, 1.0 mM Na-Pyruvate and 10 mM HEPES (pH 7.4 with NaOH). The internal solution contained 140 mM KCl, 5.0 mM NaCl, 10 mM HEPES, 5 mM Mg-ATP and 5 mM EGTA (pH 7.2 with KOH). Key action potential parameters were quantified, including maximal diastolic potential (MDP), overshoot, action potential amplitude (APA), action potential duration at 50% and 90% repolarization (APD_50_ and APD_90_), maximal upstroke velocity (V_max_), beating rate, and SD of inter-spike intervals (ISIs). Ventricular-like iPSC-CMs were distinguished based on the action potential morphology and action potential parameters, which exhibit a clear plateau phase, larger APA and V_max_ values, more negative MDP values, APD_30-40_/APD_70–80_ > 1.5 and APD_90_/APD_50_ ≤ 1.3.

Sodium current was recorded from single iPSC-CMs using the ruptured patch clamp technique with conventional voltage clamp protocols. Bath solution contained: 50 mM NaCl, 110 mM CsCl, 1.8 mM CaCl_2_, 1 mM MgCl_2_, 10 mM glucose, 10 mM HEPES and 0.001 mM nifedipine (pH 7.4 with CsOH). Pipette solutions contained: 10 mM NaCl, 135 mM CsCl, 2 mM CaCl_2_, 5 mM MgATP, 5 mM EGTA, and 10 mM HEPES (pH 7.2 with CsOH). All currents were normalized to cell capacitance to obtain current density. Steady-state activation and inactivation curves were fitted by using a Boltzmann equation: f = 1/{1 + exp[± (V − V_1/2_)/k]}, in which V_1/2_ is half-maximum (in)activation potential and k is slope factor. For analyzing time constants of inactivation, sodium currents were evoked by depolarizing cardiomyocytes from a holding potential of − 120 mV to test potentials from − 110 to − 20 mV with 10 mV increments. Recovery from inactivation of sodium currents was measured by a two-pulse protocol containing the prepulse at − 20 mV for 20 ms, then followed by various intervals before stepping to the test pulse at − 20 mV for 20 ms. Time constants (τ_fast_ and τ_slow_) of inactivation or recovery from inactivation were analyzed by curve fitting to double-exponential equation: I/I_max_ = A_fast_[1 − exp(− t/τ_fast_)] + A_slow_[1 − exp(− t/τ_slow_)], in which A_fast_ and A_slow_ are the fractions of the fast and slow components of inactivation or recovery from inactivation, and τ_fast_ and τ_slow_ are the time constants of the fast and slow components of inactivation or recovery from inactivation, respectively.

### Compounds and solutions

All the chemicals used in the electrophysiological studies were purchased from Sigma-Aldrich. CHIR99021 was purchased from Axon Medchem, and stock solutions were prepared in 10 mM in dimethyl sulfoxide (DMSO) (Sigma-Aldrich, D2650). IWR-1 was purchased from Calbiochem, and stock solutions were prepared in 10 mM in DMSO.

### Statistical analysis

Statistical significance was determined by unpaired two-tailed Student’s *t*-test to compare two groups and by One-way ANOVA to compare multiple groups. A *p* value of < 0.05 was considered statistically significant. Data were shown as mean ± sem and analyzed by GraphPad Prism (GraphPad Software).

## Results

### Clinical characteristics

**Fig. 1:**
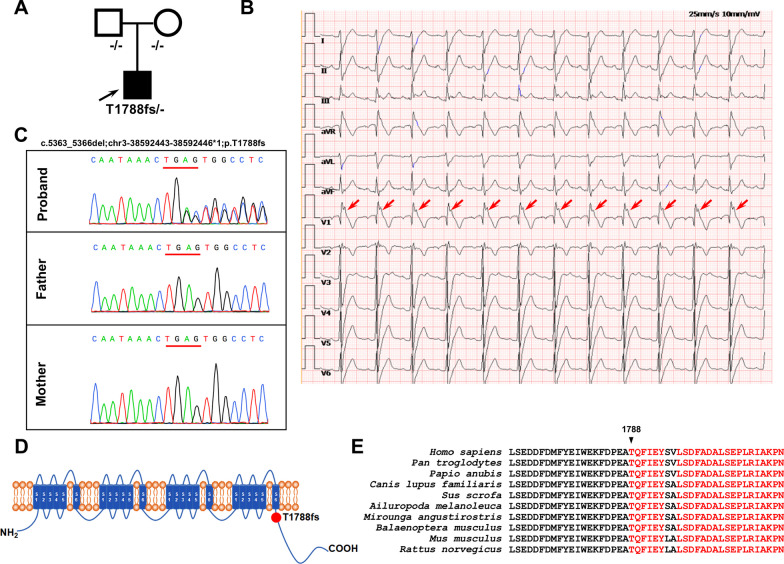
Clinical characteristics. **A** The family pedigree of the recruited patient carrying heterozygous frameshift mutation (T1788fs) in the *SCN5A* gene. Squares and circles represent male and female subjects, respectively. White and black symbols indicate unaffected and affected individuals. The arrow indicates the proband. **B** Typical electrocardiogram (ECG) from the proband demonstrating the marked type 1 BrS pattern in the lead V1 indicated by the red arrows. **C** DNA sequence chromatograms of the proband and his parents, depicting a heterozygous frameshift mutation (c.5363-5366del; p.T1788fs) in the *SCN5A* gene in the proband but not in his parents. **D** Schematic representation of the cardiac sodium channel Na_v_1.5. The identified T1788fs mutation locates at the C terminus of Na_v_1.5. **E** Sequence alignment of amino acids adjacent to Thr1788 in the *SCN5A* gene among 10 different species

One BrS patient and three healthy control subjects were recruited in this study (Fig. 1A and Additional file 1: Table S2). The patient presented to the local hospital complaining of palpitations, amaurosis and recurrent syncope at 24 years old. His resting ECG exhibited a classical type 1 Brugada pattern (Fig. 1B), and echocardiography showed normal heart structure (Additional file 1: Figure S1). He had no family history of heart disease. Subsequently, he was clinically diagnosed with BrS and received ICD implantation because of a history of recurrent syncope. However, in the following 2 years, palpitations and amaurosis still recurred frequently, which were relieved after ICD shock. Because the ICD recorded episodes of ventricular tachycardia and fibrillation, a cardiac electrophysiological study and catheter ablation were performed 2 years later. During the 7-year follow-up period after catheter ablation, the patient had no significant complaints. Genetic testing identified a heterozygous mutation of 4-base-pair deletion in the *SCN5A* gene (c.5363-5366del; p.T1788fs), which results in a frameshift starting at amino acid 1788 (Fig. 1C). In contrast, no *SCN5A* mutation was detected in his parents (Fig. 1C). The mutation site is located at the C-terminus of Na_v_1.5 and leads to the premature termination of protein translation (Fig. 1D). As shown in Fig. 1E, the mutation region is highly conserved among different species. Because this is a de novo* SCN5A* mutation in the patient (both paternity and maternity confirmed), and this mutation was not found in controls in Exome Sequencing Project, 1000 Genomes Project, or Exome Aggregation Consortium, the pathogenic level of this novel *SCN5A* mutation was therefore classified as “likely pathogenic” according to the American College of Medical Genetics and Genomics (ACMG) guidelines (PS2 + PM2) (PS: pathogenic strong; PM: pathogenic moderate) [32].

### Generation and characterization of patient-specific iPSC-CMs

**Fig. 2:**
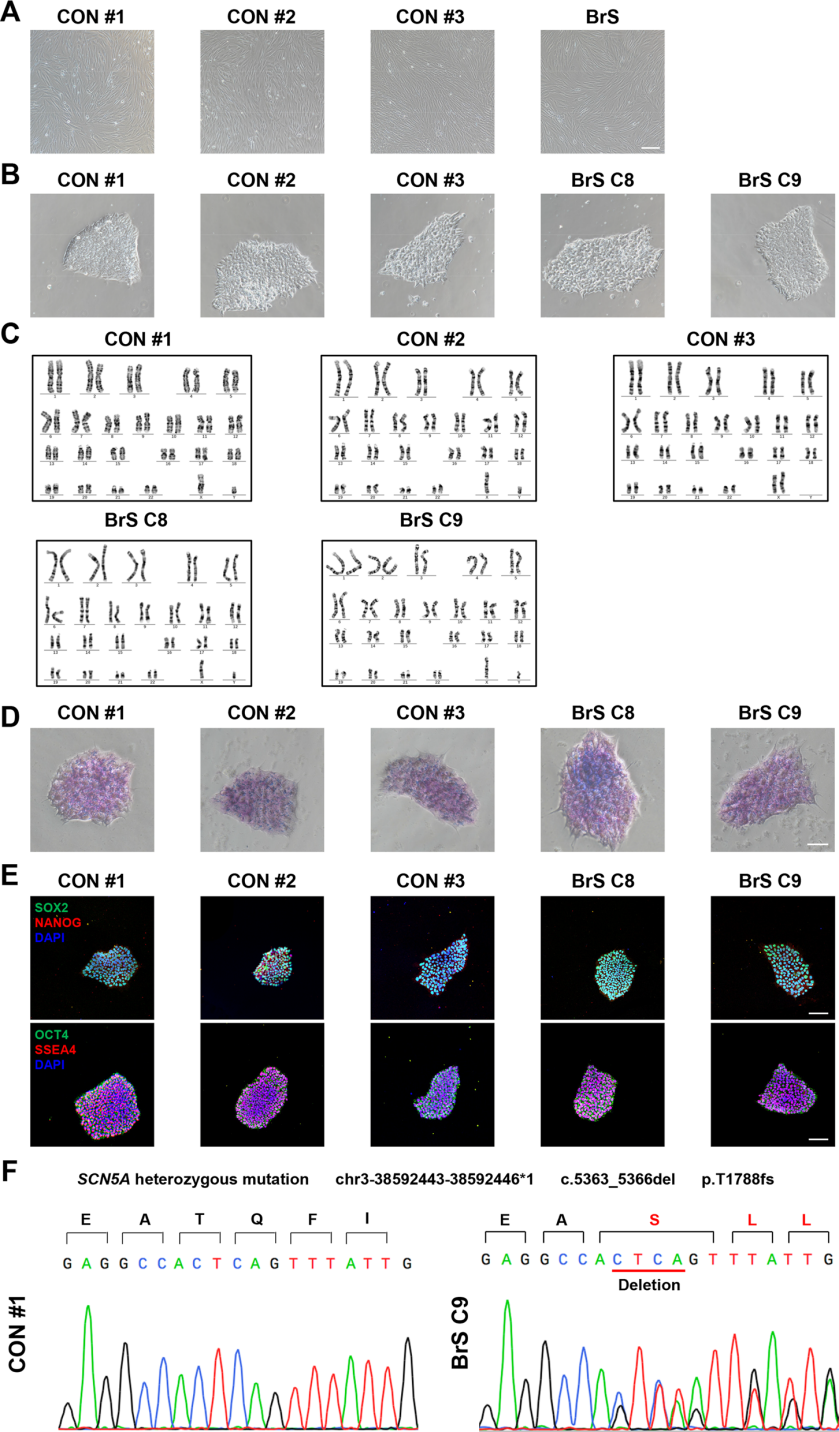
Generation and characterization of iPSC-CMs. **A** Typical morphology of skin fibroblasts from the healthy control subjects and the BrS patient. Scale bar, 400 μm. **B** Typical morphology of control and BrS iPSCs. Scale bar, 100 μm. **C.** Representative graphs of karyotypes of control and BrS iPSCs. **D** Representative graphs of ALP staining of control and BrS iPSCs. Scale bar, 100 μm. **E** Representative graphs of pluripotent staining of control and BrS iPSCs using SOX2 (green), NANOG (red), OCT4 (green) and SSEA4 (red). DAPI indicates nuclear staining (blue). Scale bar, 100 μm. **F** Confirmation of the existence of the *SCN5A* T1788fs mutation in BrS iPSCs (BrS C9 iPSC line) but not in control iPSCs (CON#1 iPSC line)

Skin biopsies were obtained from the BrS patient and healthy control subjects. The skin fibroblasts were reprogrammed using the Sendai viral method to generate iPSCs (Fig. 2A). The successfully generated iPSCs exhibited typical human embryonic stem cell-like morphology and normal karyotypes (Fig. 2B, C), stained positively for alkaline phosphatase (ALP) and pluripotency markers (SOX2, NANOG, OCT4 and SSEA4) (Fig. 2D, E and Additional file 1: Figure S2). Sanger sequencing confirmed that the *SCN5A* T1788fs mutation existed in patient iPSCs but not in control iPSCs (Fig. 2F and Additional file 1: Figure S3). Control and BrS iPSCs were subsequently differentiated into cardiomyocytes using a small molecule-based monolayer protocol as previously described [33, 34]. The generated iPSC-CMs demonstrated positive staining for cardiac-specific markers TNNT2 and α-actinin (Additional file 1: Figure S4).

### BrS iPSC-CMs carrying *SCN5A* T1788fs exhibit Na_v_1.5 defects and an arrhythmic phenotype

**Fig. 3:**
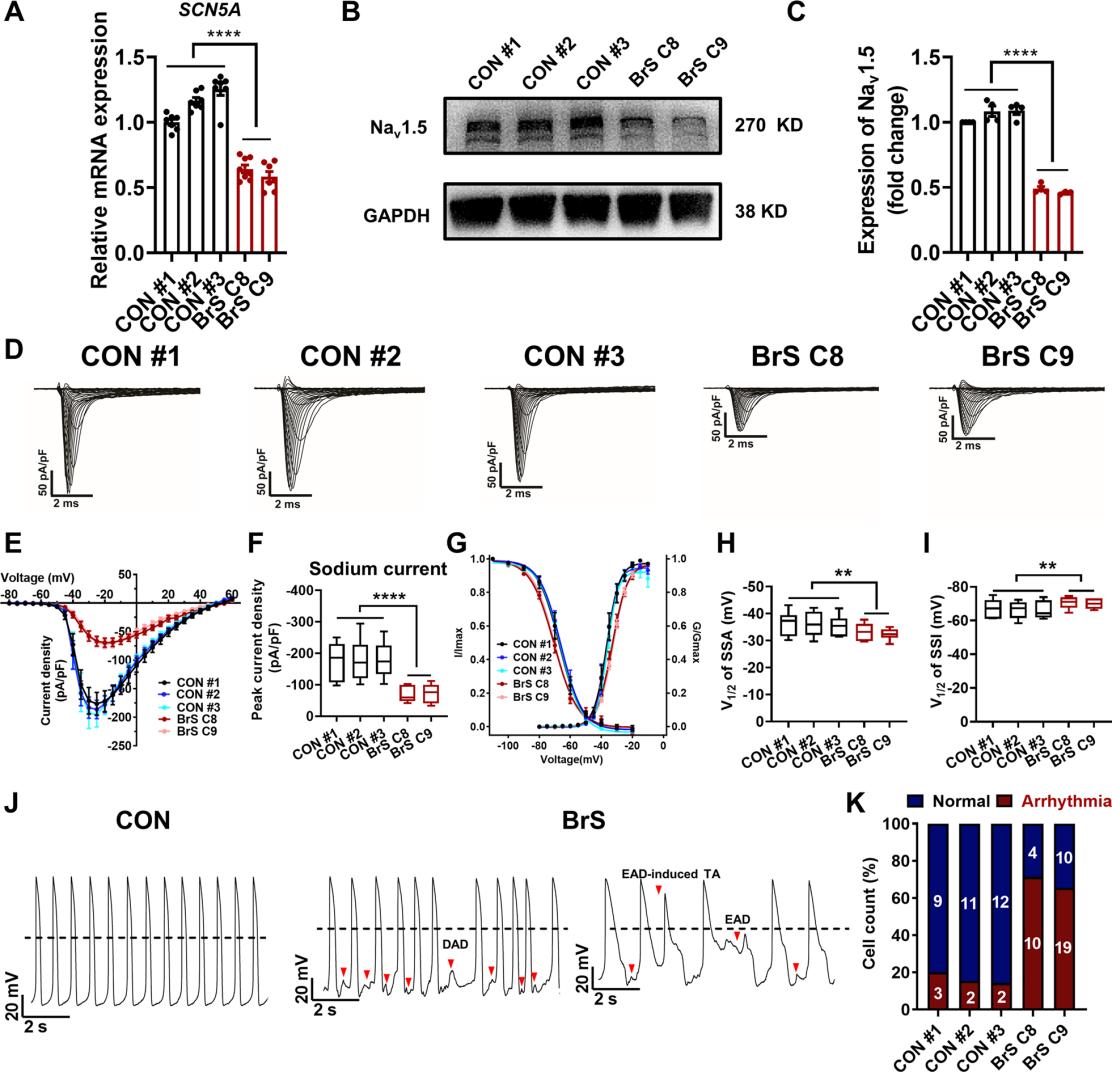
BrS iPSC-CMs carrying *SCN5A* T1788fs exhibit Na_v_1.5 defects and arrhythmic phenotypes.** A** Bar graph to compare the mRNA expression of *SCN5A* between control (CON) and BrS iPSC-CMs by qPCR. *n* = 7–8 technical replicates. ****p < 0.0001. **B** Western blot analysis of Na_v_1.5 expression in control and BrS iPSC-CMs. Full-length blots are presented in Additional file 1: Fig. S5. **C** Bar graph to compare the Na_v_1.5 expression between control and BrS iPSC-CMs. *n* = 3–4 culture replicates. ****p < 0.0001. **D** Representative sodium current tracings isolated from control and BrS iPSC-CMs. **E** Comparison of IV curve of sodium current between control and BrS iPSC-CMs. **F** Bar graph to compare peak sodium current density at − 20 mV between control and BrS iPSC-CMs. *n* = 8 cells. ****p < 0.0001. **G** Comparison of steady-state activation (SSA) and steady-state inactivation (SSI) of sodium current between control and BrS iPSC-CMs. **H**, **I** Bar graphs to compare V_1/2_ of SSA and SSI of sodium current between control and BrS iPSC-CMs. **p < 0.01. **J** Representative action potential tracings recorded by single-cell patch clamp from control and BrS ventricular-like myocytes. Dashed lines indicate 0 mV. Red arrows indicate putative arrhythmias in BrS iPSC-CMs. **K** Bar graph to compare the percentage of iPSC-CMs with arrhythmias between control and BrS iPSC-CMs. *n* = 12–29 cells

We next sought to assess whether the T1788fs mutation relates to the expression of *SCN5A* and its encoding protein Na_v_1.5. We observed significantly reduced expression of *SCN5A* and Na_v_1.5 at mRNA and protein levels in BrS iPSC-CMs, when compared to control counterparts (Fig. 3A–C, Additional file 1: Figure S5 and Table S1). To investigate if the expression changes may give rise to functional consequences, we isolated sodium channel currents from control and BrS iPSC-CMs by single-cell patch clamp recordings. BrS iPSC-CMs exhibited dramatically reduced sodium current density in comparison with controls (Fig. 3D–F). In addition, the steady-state activation (SSA) curve of the sodium channel in BrS iPSC-CMs was positively shifted by 3.33 mV as compared to controls, whereas the steady-state inactivation (SSI) curve was negatively shifted by 3.67 mV (Fig. 3G–I). Time constants of inactivation of sodium currents at most of the tested voltages were comparable between control and BrS iPSC-CMs, while *τ*_fast_ at − 30 mV and − 25 mV, and *τ*_slow_ at − 25 mV and − 20 mV were significantly increased in BrS iPSC-CMs (Additional file 1: Figure S6A–C). Analysis of recovery from inactivation of sodium currents revealed that there was no significant change of *τ*_fast_ between control and BrS iPSC-CMs, whereas *τ*_slow_ and fractional *A*_fast_ were significantly reduced in BrS iPSC-CMs (Additional file 1: Figure S6D–G). Moving forward, single-cell action potentials were recorded from control and BrS ventricular-like myocytes, and key action potential parameters were quantified. We observed normal and rhythmic action potential patterns in control iPSC-CMs, while a large proportion of BrS iPSC-CMs demonstrated arrhythmic action potential waveforms, manifesting as delayed afterdepolarizations (DADs), early afterdepolarizations (EADs) and EAD-induced triggered activities (TAs) (Control: 17.9%; BrS: 67.4%) (Fig. 3J, K). Moreover, we observed significantly increased peak-peak interval variability, decreased action potential amplitude (APA) and maximal upstroke velocity (V_max_) in BrS iPSC-CMs, whereas beating rate and action potential duration (APD) were comparable between control and BrS iPSC-CMs (Additional file 1: Figure S7). Taken together, these results indicate Na_v_1.5 defects and arrhythmic phenotype in BrS iPSC-CMs carrying *SCN5A* T1788fs.

### Activation of Wnt/β-catenin signaling suppresses Na_v_1.5 expression and function in iPSC-CMs

**Fig. 4:**
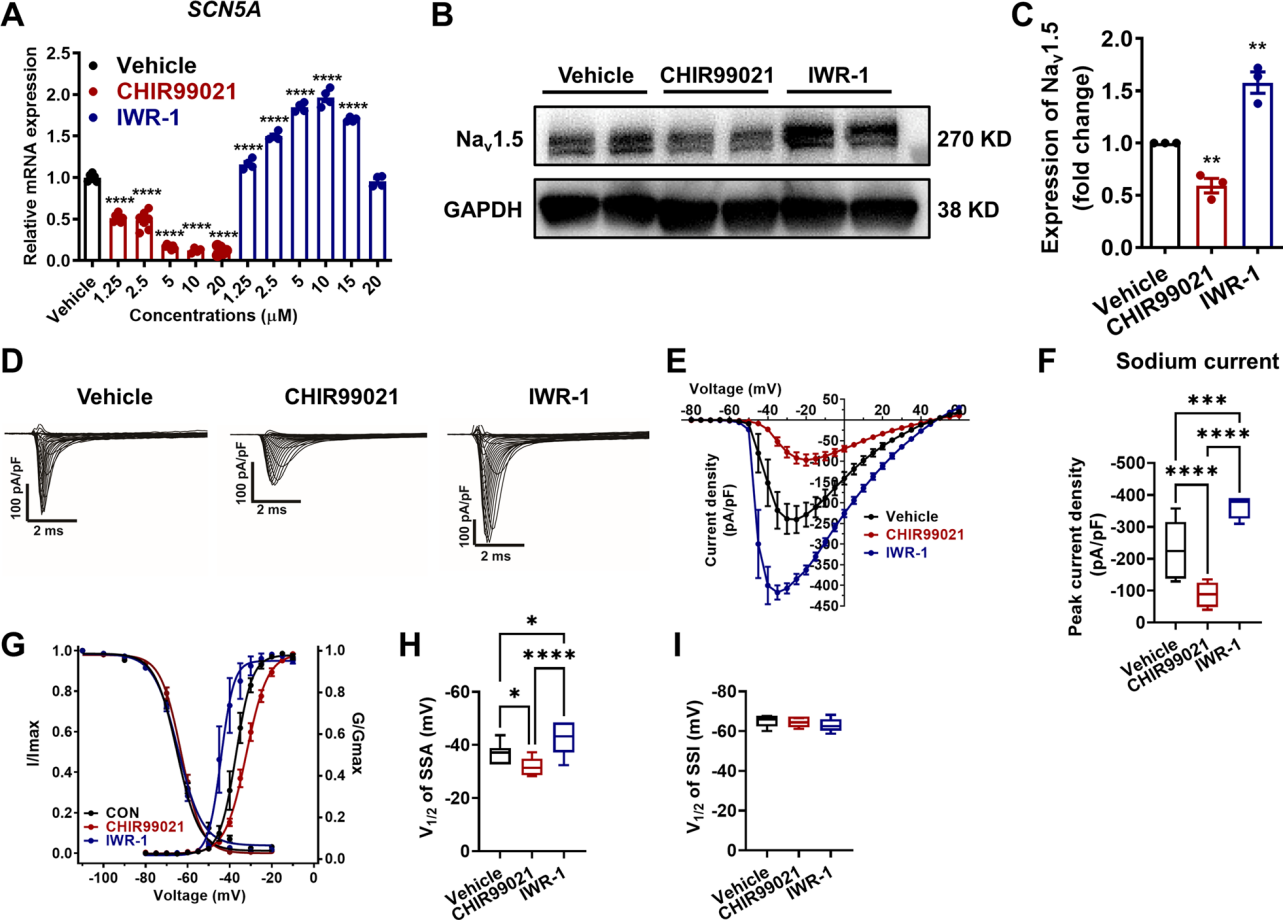
Activation of Wnt/β-catenin signaling suppresses cardiac Na^ +^ channel Na_v_1.5 expression and function in iPSC-CMs. **A** Bar graph to compare the mRNA expression of *SCN5A* in control iPSC-CMs after treatment with CHIR99021 (1.25, 2.5, 5, 10 and 20 μM) or IWR-1 (1.25, 2.5, 5, 10, 15 and 20 μM) for 48 h, respectively. *n* = 4–7 technical replicates. ****p < 0.0001. **B** Western blot analysis of Na_v_1.5 expression in control iPSC-CMs treated with DMSO only (vehicle), 5 μM CHIR99021 or 10 μM IWR-1 for 72 h, respectively. Full-length blots are presented in Additional file 1: Fig. S8. **C** Bar graph to compare the Na_v_1.5 expression between three different groups. *n* = 3 culture replicates. *p < 0.05 and **p < 0.01. **D** Representative sodium current tracings recorded from three different groups. **E** Comparison of IV curve of sodium current between three different groups. **F** Bar graph to compare peak sodium current density at -20 mV between three different groups. *n* = 7–11 cells. ***p < 0.001 and ****p < 0.0001. **G** Comparison of SSA and SSI of sodium current between three different groups. **H**, **I** Bar graphs to compare V_1/2_ of SSA and SSI of sodium current between three different groups. *n* = 7–14 cells. *p < 0.05

Previous studies have reported that activation of Wnt/β-catenin signaling suppresses Na_v_1.5 expression in HL-1 cells and neonatal rat ventricular myocytes [12–15]. Consistently, we observed that treatment of CHIR99021, a specific GSK-3 inhibitor to effectively activate Wnt signaling, resulted in significantly reduced expression of *SCN5A* and Na_v_1.5 (Fig. 4A–C and Additional file 1: Figure S8), reduced sodium current density (Fig. 4D–F), and a right-shift of the SSA curve (Fig. 4G, H) in control iPSC-CMs. In contrast, treatment of IWR-1 in control iPSC-CMs, a specific Wnt/β-catenin signaling inhibitor, markedly enhanced the cardiac sodium channel Na_v_1.5 expression and function (Fig. 4A–I). Collectively, these results indicate that activation of Wnt/β-catenin signaling suppresses Na_v_1.5 expression and function in iPSC-CMs.

### Aberrant activation of Wnt/β-catenin signaling in BrS iPSC-CMs

**Fig. 5:**
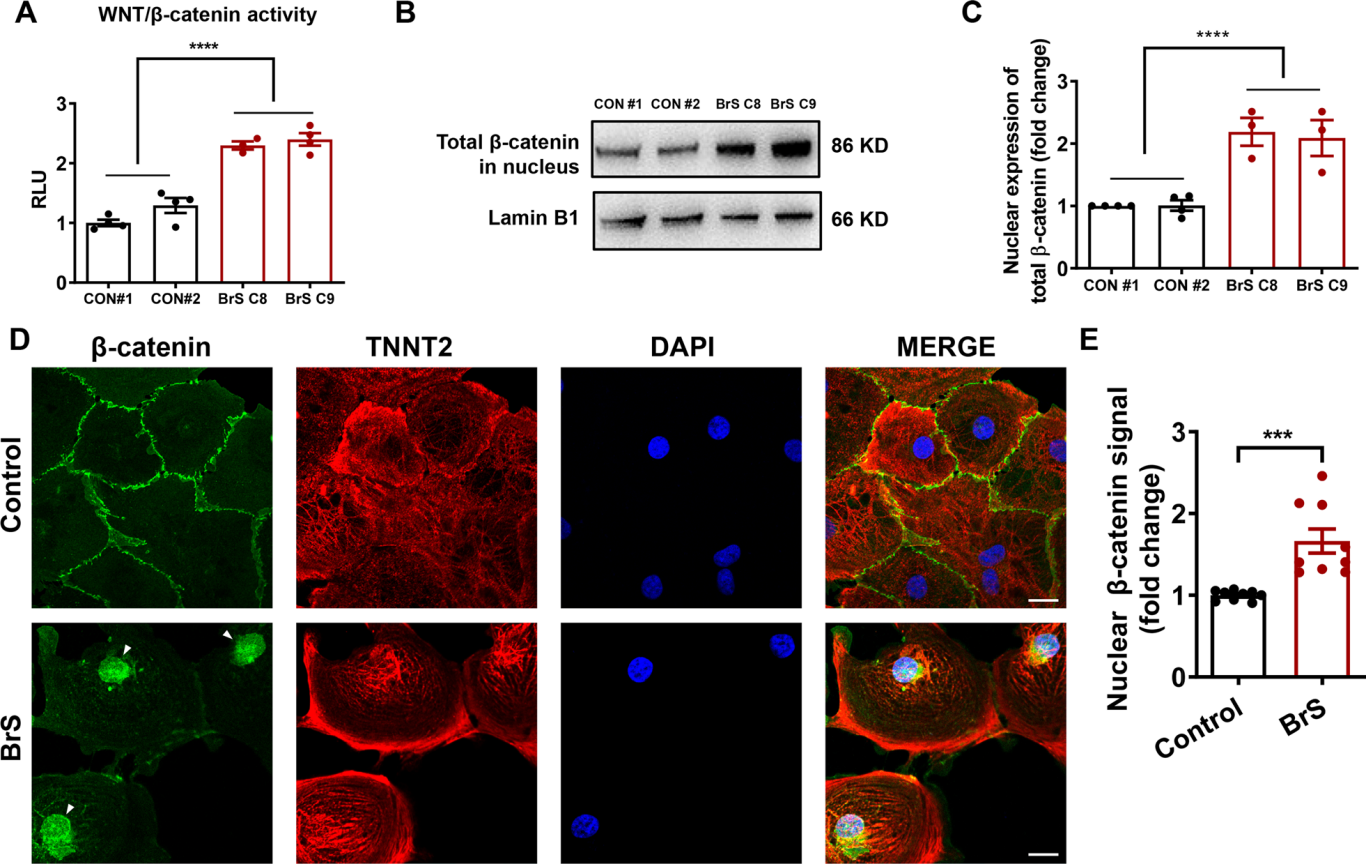
Aberrant activation of Wnt/β-catenin in BrS iPSC-CMs. **A** Bar graph to compare the Wnt/β-catenin signaling activity between control and BrS iPSC-CMs evaluated by the TOPflash reporter assay. *n* = 3–4 culture replicates. ****p < 0.0001. **B** Western blot analysis of the nucleus expression of total β-catenin in control and BrS iPSC-CMs. Lamin B1 is used for the loading control. Full-length blots are presented in Additional file 1: Fig. S9. **C** Bar graph to compare the nucleus expression of β-catenin in control and BrS iPSC-CMs. *n* = 3–4 culture replicates. ****p < 0.0001. **D** Representative confocal images of β-catenin (green) staining in control and BrS iPSC-CMs, showing increased nuclear localization of β-catenin in BrS iPSC-CMs. TNNT2 (red) is used as a specific marker of cardiomyocytes. DAPI indicates nuclear staining (blue). White arrows indicate the nuclear localization of β-catenin in BrS iPSC-CMs. Scale bar, 20 μm. **E** Bar graph to compare the signal intensity of nuclear versus cytoplasmic β-catenin from the confocal images in C. *n* = 9 views. ***p < 0.001

Given the cardiac sodium channel defects seen in BrS iPSC-CMs, we next performed TOPflash Wnt reporter assay to assess the level of Wnt/β-catenin signaling in control and BrS iPSC-CMs. The results revealed markedly increased Wnt/β-catenin activity in BrS iPSC-CMs in comparison with controls (Fig. 5A). The accumulation of β-catenin protein in the nucleus is a key indicator of Wnt/β-catenin signaling activation [35]. Western blot analysis revealed greatly higher levels of total and active form of nuclear β-catenin expression in BrS iPSC-CMs than those in control iPSC-CMs (Fig. 5B, C and Additional file 1: Figure S9–S11). Moreover, control and BrS iPSC-CMs were double-stained with β-catenin and TNNT2 antibodies, and significantly stronger signals of nuclear β-catenin were detected in BrS iPSC-CMs compared to controls, pointing to the activation of Wnt/β-catenin signaling (Fig. 5D, E).

### Inhibition of Wnt/β-catenin rescues Na_v_1.5 defects and arrhythmic phenotype in BrS iPSC-CMs

**Fig. 6:**
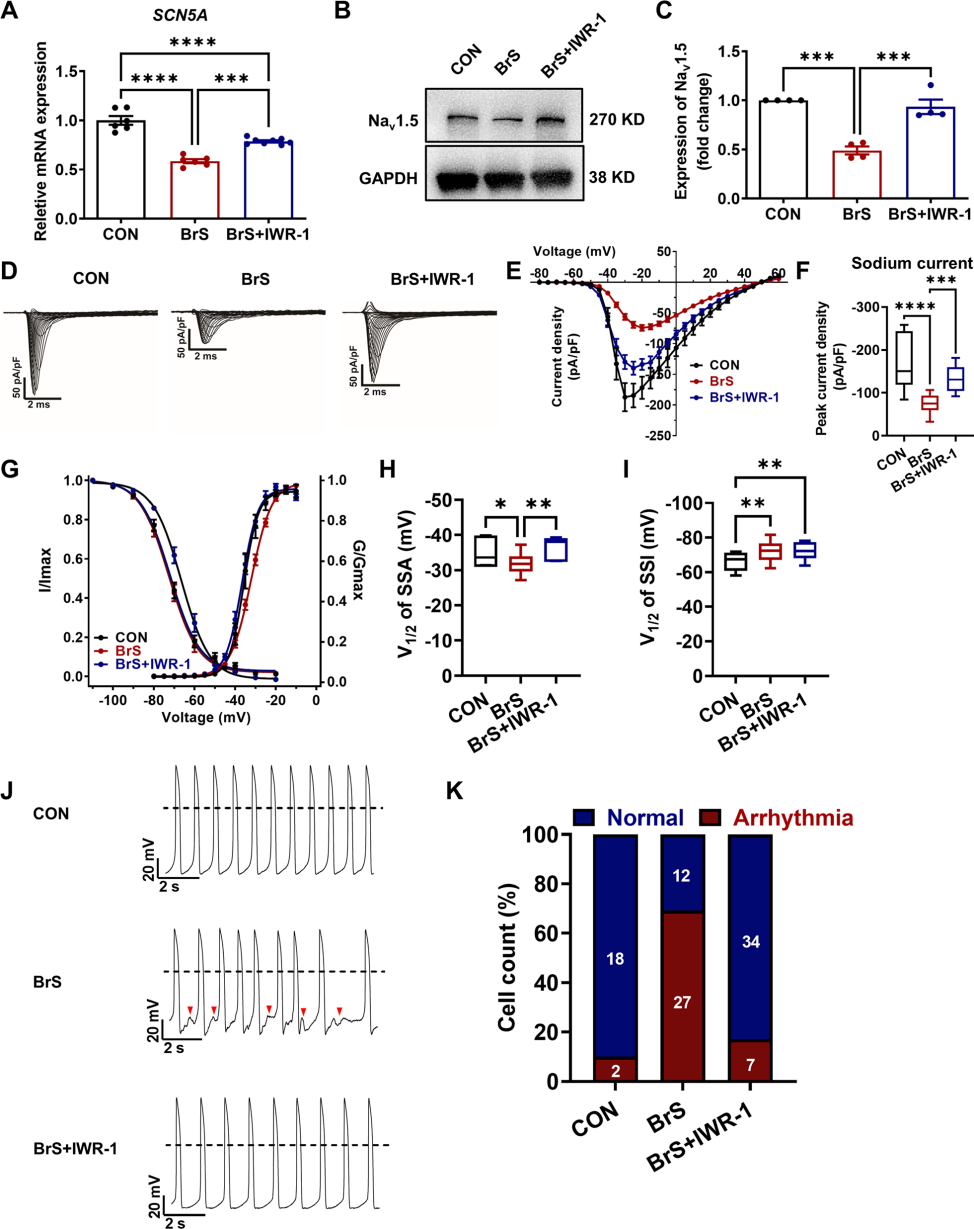
Inhibition of Wnt/β-catenin rescues Na^ +^ channel defects and arrhythmic phenotypes in BrS iPSC-CMs. A. Bar graph to compare the mRNA expression of *SCN5A* in control iPSC-CMs, BrS iPSC-CMs treated with DMSO only, and BrS iPSC-CMs treated with 10 μM IWR-1. *n* = 6–8 technical replicates. ***p < 0.001 and ****p < 0.0001. **B** Western blot analysis of Na_v_1.5 expression in three different groups. Full-length blots are presented in Additional file 1: Fig. S12. **C** Bar graph to compare the Na_v_1.5 expression between three different groups. *n* = 4 culture replicates. ***p < 0.001. **D** Representative sodium current tracings recorded from three different groups. **E** Comparison of IV curve of sodium current between three different groups. **F** Bar graph to compare peak sodium current density at − 20 mV between three different groups. *n* = 10–17 cells. ***p < 0.001 and ****p < 0.0001. **G** Comparison of SSA and SSI of sodium current between three different groups. **H**, **I** Bar graphs to compare V_1/2_ of SSA and SSI of sodium current between three different groups. *n* = 8–18 cells. *p < 0.05 and **p < 0.01. **J** Representative action potential tracings recorded by single-cell patch clamp from three different groups. Dashed lines indicate 0 mV. Red arrows indicate putative arrhythmias in BrS iPSC-CMs treated with DMSO. **K** Bar graph to compare the percentage of iPSC-CMs with arrhythmias between three different groups. *n* = 20–41 cells

We next assessed whether inhibition of Wnt/β-catenin could ameliorate the abnormalities seen in BrS iPSC-CMs. Treatment of IWR-1 in BrS iPSC-CMs significantly rescued the expression of both *SCN5A* and Na_v_1.5, when compared to treatment of DMSO only (vehicle) (Fig. 6A–C and Additional file 1: Figure S12). We also observed largely improved sodium channel function in IWR-1-treated BrS iPSC-CMs as compared to DMSO-treated BrS iPSC-CMs, demonstrating restored sodium current density and SSA curve (Fig. 6D–I). Importantly, treatment of IWR-1 greatly attenuated the arrhythmias observed in BrS iPSC-CMs, resembling the normal action potential pattern of control iPSC-CMs (Fig. 6J, K and Additional file 1: Figure S13). These results indicate that inhibition of Wnt/β-catenin signaling can rescue both the Na_v_1.5 defects and the arrhythmic phenotype in BrS iPSC-CMs.

### Na_v_1.5 interacts with β-catenin

**Fig. 7:**
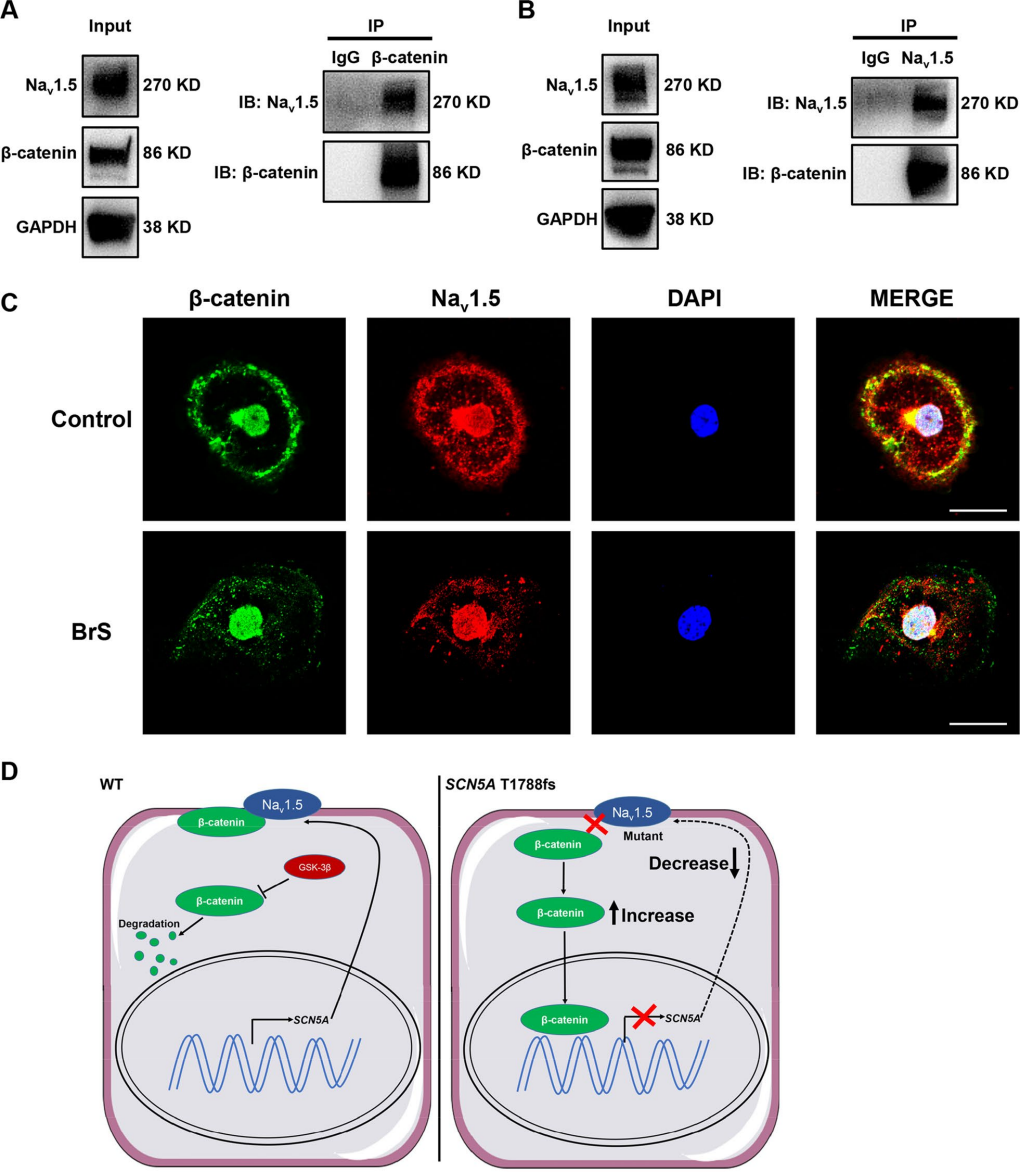
Na_v_1.5 interacts with β-catenin. **A** Na_v_1.5 was detected in the immunoprecipitation pull-down lysate from control iPSC-CMs using antibody against β-catenin, indicating that Na_v_1.5 interacts with β-catenin. IP: β-catenin, IP with β-catenin antibody. Full-length blots are presented in Additional file 1: Fig. S14. **B** β-catenin was detected in the immunoprecipitation pull-down lysate from control iPSC-CMs using antibody against Na_v_1.5, indicating that β-catenin interacts with Na_v_1.5. IP: Na_v_1.5, IP with Nav1.5 antibody. Full-length blots are presented in Additional file 1: Fig. S15. **C** Representative confocal images of β-catenin (green) and Nav1.5(red) staining in control and BrS iPSC-CMs, showing the co-localization of Nav1.5 and β-catenin in iPSC-CMs. Scale bar, 20 μm. **D** Schematic representation of the proposed mechanism of *SCN5A*-T1788fs action in cardiomyocytes. Left: In control iPSC-CMs, β-catenin is mainly localized in the cell membrane with Na_v_1.5. Cytoplasmic β-catenin is at a low level and is degraded by the destruction complex. Right: In BrS iPSC-CMs carrying the *SCN5A*-T1788fs mutation, the loss of Na_v_1.5 disturbs the distribution of β-catenin and subsequently increases nuclear localization of β-catenin, which leads to the suppression of *SCN5A* transcription. Thus, a vicious circle is formed to cause arrhythmias in mutant myocytes

We next sought to investigate the molecular mechanisms underlying the aberrant activation of Wnt/β-catenin signaling in BrS iPSC-CMs. A previous study reported that knockdown of Na_v_1.5 affected the expression of β-catenin [36]. We therefore reasoned that a protein–protein interaction may exist between Na_v_1.5 and β-catenin. To test this hypothesis, co-immunoprecipitation (co-IP) studies were conducted to test whether there was an interaction between Na_v_1.5 and β-catenin in iPSC-CMs. Notably, reciprocal co-precipitation of Na_v_1.5 and β-catenin was observed using respective antibodies (Fig. 7A, B and Additional file 1: Figure S14–S15). Moreover, immunofluorescent staining revealed colocalization of Na_v_1.5 and β-catenin in iPSC-CMs (Fig. 7C). Interestingly, accompanied by the Na_v_1.5 defects in BrS iPSC-CMs, a markedly different distribution of β-catenin was observed between control and BrS iPSC-CMs. Considerable amounts of β-catenin were detected in the nuclei of BrS iPSC-CMs, whereas β-catenin was mainly found in the cell membrane of control iPSC-CMs, suggesting that the loss of Na_v_1.5 may affect the distribution of β-catenin in BrS iPSC-CMs (Fig. 7C, D). Altogether, these results suggest that an interaction between Na_v_1.5 and β-catenin exists, and reduced expression of Na_v_1.5 leads to the redistribution of β-catenin from the plasma membrane to the nucleus in BrS iPSC-CMs.

## Discussion

Here, we utilized a patient-specific iPSC-CM model to investigate the role of Wnt/β-catenin signaling in *SCN5A*-related BrS and underlying molecular mechanisms.

Human iPSC lines were successfully generated from healthy control subjects and a BrS patient carrying a novel frameshift mutation (c.5363-5366del; p.T1788fs) in the *SCN5A* gene. In line with previous studies [24, 26], analysis of BrS iPSC-CMs revealed an increased burden of arrhythmias and cardiac sodium channel Na_v_1.5 defects as compared to control iPSC-CMs. Interestingly, these functional abnormalities observed in BrS iPSC-CMs were accompanied by the aberrant activation of Wnt/β-catenin signaling. Moreover, we found that inhibition of Wnt/β-catenin significantly rescued Na_v_1.5 defects and arrhythmic phenotype in BrS iPSC-CMs. Mechanistically, *SCN5A*-encoded Na_v_1.5 interacts with β-catenin, and reduced expression of Na_v_1.5 leads to re-localization of β-catenin in BrS iPSC-CMs, which aberrantly activates Wnt/β-catenin signaling to suppress *SCN5A* transcription.

Wnt/β-catenin signaling is involved in various physiological and pathological activities [11]. When this pathway remains inactivated, cytoplasmic β-catenin as the downstream factor of canonical Wnt signaling was degraded by the “destruction complex” which includes APC, Axin, CK1 and GSK3 [37]. When the Wnt signaling is activated, the function of the “β-catenin destruction complex” is inhibited, leading to the stabilization of β-catenin. The stabilized β-catenin accumulates in the cytoplasm and then, transfers to the nucleus, regulating the expression of kinds of genes [38]. As mentioned above, aberrant activation of Wnt/β-catenin signaling is discovered in various heart diseases and alternation of Wnt/β-catenin activity was recently observed in inherited heart diseases [16–18, 20]. It has been reported that loss of cardiac plakoglobin leads to the up-regulation of Wnt/β-catenin signaling activity [16]. In addition, filamin C deficiency affects the localization of β-catenin and results in the activation of Wnt/β-catenin signaling [20]. However, the association between Wnt/β-catenin signaling and *SCN5A*-related BrS, featured by the loss-of-function of Na_v_1.5, is still unknown. In our work, a novel and important finding is the aberrant activation of Wnt/β-catenin signaling in BrS iPSC-CMs, which has not been previously reported.

Previous studies have shown that activation of Wnt/β-catenin signaling inhibits the transcription of *SCN5A*, resulting in the reduction in sodium current [12, 13]. Wnt/β-catenin signaling mediates *SCN5A* transcription in both direct and indirect ways, which has been elucidated using neonatal rat ventricular myocytes [14]. Briefly, the β-catenin/TCF4 complex can directly bind to the promoter of *SCN5A* and subsequently inhibit the transcription. This complex can also increase the expression of *Tbx3*, a known suppressor of *SCN5A*. Although the regulation role of Wnt/β-catenin signaling on the expression of *SCN5A* is known, we found an unexpected phenomenon that the level of Na_v_1.5 can in turn affect the activity of Wnt/β-catenin signaling. To our knowledge, we demonstrated the interaction between Na_v_1.5 and β-catenin for the first time, and we suggest this novel link contributes to the redistribution of β-catenin in BrS iPSC-CMs with reduced expression of Na_v_1.5.

It has been known that both the morbidity and mortality of BrS are high [3]. Nevertheless, treatments for BrS are few at present [5]. Consequently, finding an effective treatment strategy is urgent and meaningful. Given the findings of aberrant activation of Wnt/β-catenin signaling in BrS iPSC-CMs, we tested if inhibition of such signaling could alleviate the cellular arrhythmic phenotype in BrS. Since there are no Food and Drug Administration (FDA)-approved Wnt signaling inhibitors at present [39], IWR-1 as a widely used small-molecule inhibitor of Wnt/β-catenin signaling was chosen. The results of rescue experiments employing IWR-1 for blocking Wnt signaling revealed recovery of disease phenotypic features in BrS iPSC-CMs. Our findings therefore suggest that inhibition of Wnt/β-catenin could present a potential therapeutic strategy for BrS. It is worth mentioning that our work is also the first study that identified the regulation role of Wnt/β-catenin signaling on the expression of *SCN5A* in the iPSC-CM model.

There are several limitations to our work that warrant caution and future work. First, Wnt signaling pathway can be classified as β-catenin-dependent pathway (canonical Wnt signaling) and β-catenin-independent pathway (non-canonical Wnt signaling) [11]. In this work, we focused our study on canonical Wnt signaling and did not explore the role of non-canonical Wnt signaling in BrS iPSC-CMs. Similar to canonical Wnt signaling, non-canonical Wnt signaling also plays an important role in cardiovascular diseases, involving atherosclerosis, fibrosis, hypertrophy and oxidative stress [40–44]. More importantly, a complex crosstalk between the canonical and non-canonical Wnt signaling has been described [45, 46]. Therefore, while our data suggest canonical Wnt signaling may exert essential effect in the pathogenesis of BrS, non-canonical Wnt signaling may also play an important role in BrS and may be a potential confounding factors in our study. However, at present, very little is known about the function of non-canonical Wnt signaling in the pathophysiological processes of BrS, and warrants future investigation. Second, only the iPSC-CMs model was applied in this study. We consider it is very meaningful to investigate the role of Wnt signaling in BrS at the organ level in future work. An interesting point that is the Wnt-mediated developmental processes is different between right and left ventricles [47]. Meanwhile, BrS is found mainly affecting the right ventricle [48, 49]. Therefore, in our view, the study on Wnt signaling in BrS at the organ level may provide new findings and even increase our understanding of the pathogenesis of BrS. Third, it is noteworthy that widespread inhibition of the Wnt/β-catenin pathway may affect the function of other organs [50, 51]. Thus, a focus for future studies could be exploring a selective and cardiac-specific Wnt/β-catenin signaling inhibition regiment for treatment strategies in BrS patients.

## Conclusions

In conclusion, our findings suggest that aberrant activation of Wnt signaling contributes to the pathogenesis of *SCN5A*-related BrS and point to Wnt/β-catenin as a potential therapeutic target.

### Supplementary Information

**Additional file 1**. Supplemental Material.

## Data Availability

All data supporting the conclusions of this article are included within the article.

## Abbreviations

BrS: Brugada syndrome
ECG: Electrocardiogram
SCD: Sudden cardiac death
VF: Ventricular fibrillation
ICD: Implantable cardioverter-defibrillation
Na_v_1.5: Voltage-gated cardiac sodium channel
iPSCs: Induced pluripotent stem cells
iPSC-CMs: Induced pluripotent stem cell-derived cardiomyocytes
