# Supplementary material for: Patient-specific iPSC-derived cardiomyocytes reveal aberrant activation of Wnt/β-catenin signaling in SCN5A-related Brugada syndrome
Source: Stem Cell Res Ther. 2023 Sep 8;14:241. doi: 10.1186/s13287-023-03477-3 (PMC10486057; doi:10.1186/s13287-023-03477-3)
Supplement: Supplementary file 1 — Additional file 1. Supplemental Material. [file 13287_2023_3477_MOESM1_ESM.pdf]

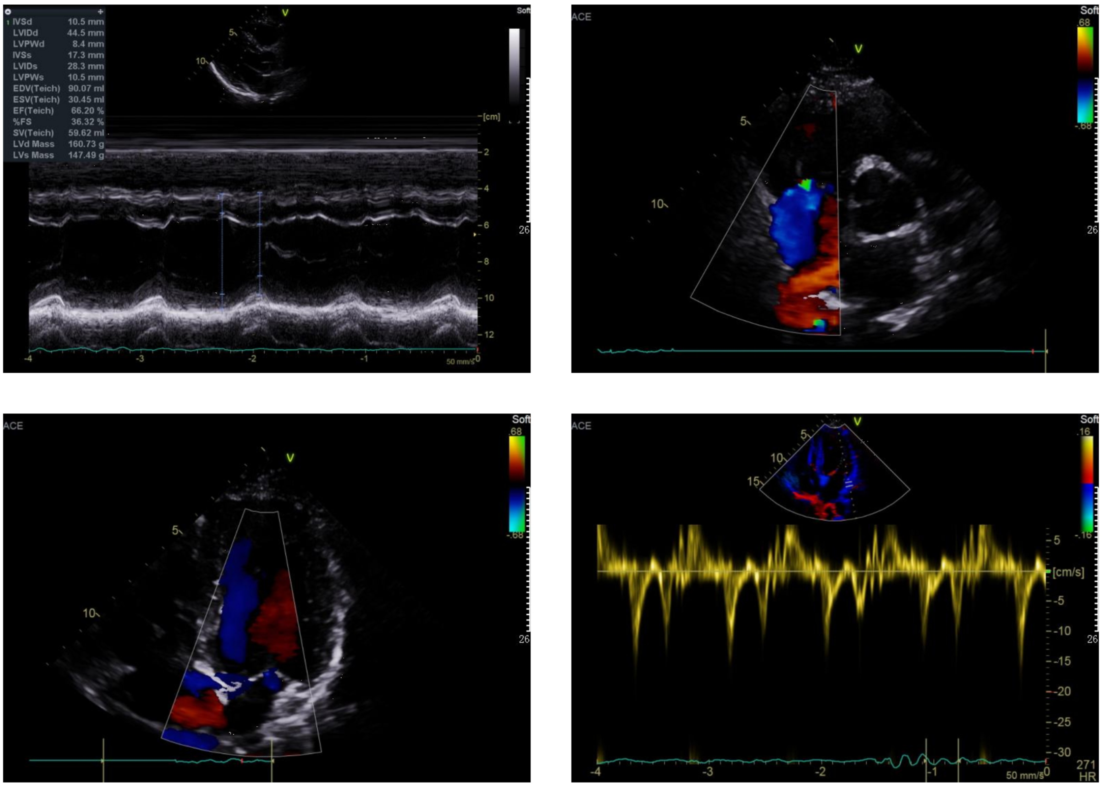

Supplemental Figure 1. Morphological characteristics of the proband. Echocardiography shows normal heart structure, apart from slight valve regurgitation.

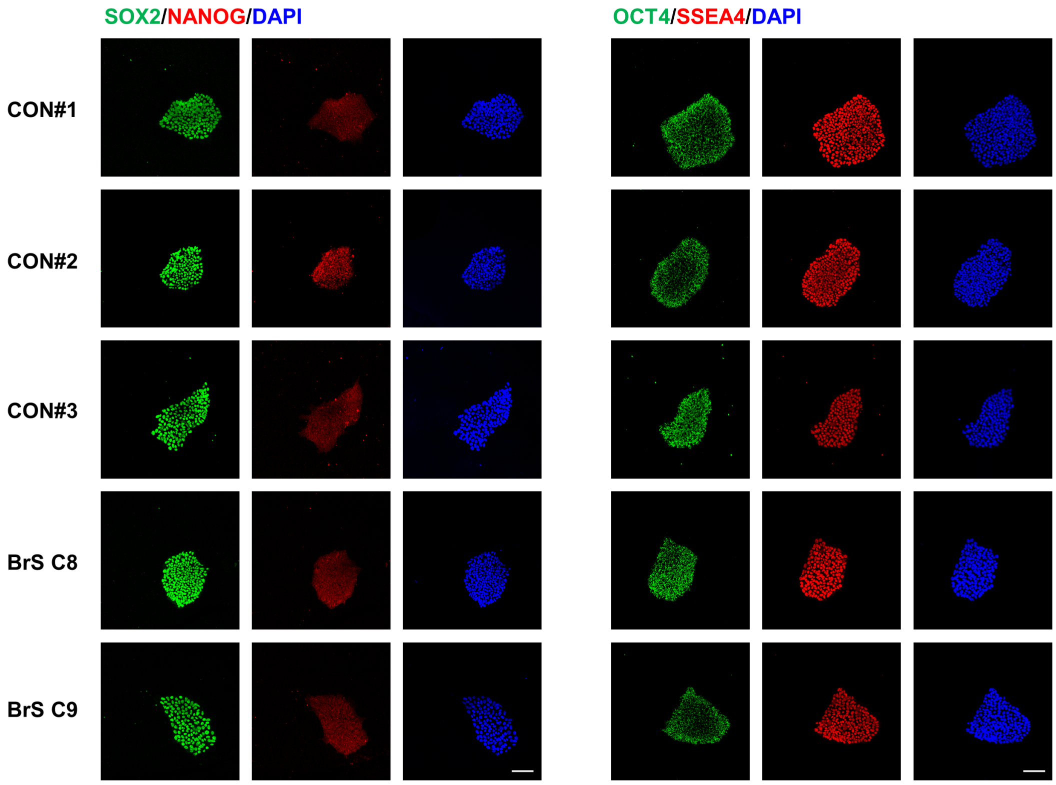

Supplemental Figure 2. Pluripotent staining of iPSCs. Representative graphs of pluripotent staining of control and BrS iPSCs using SOX2 (green), NANOG (red), OCT4 (green) and SSEA4 (red). DAPI indicates nuclear staining (blue). Scale bar, 100 µm.

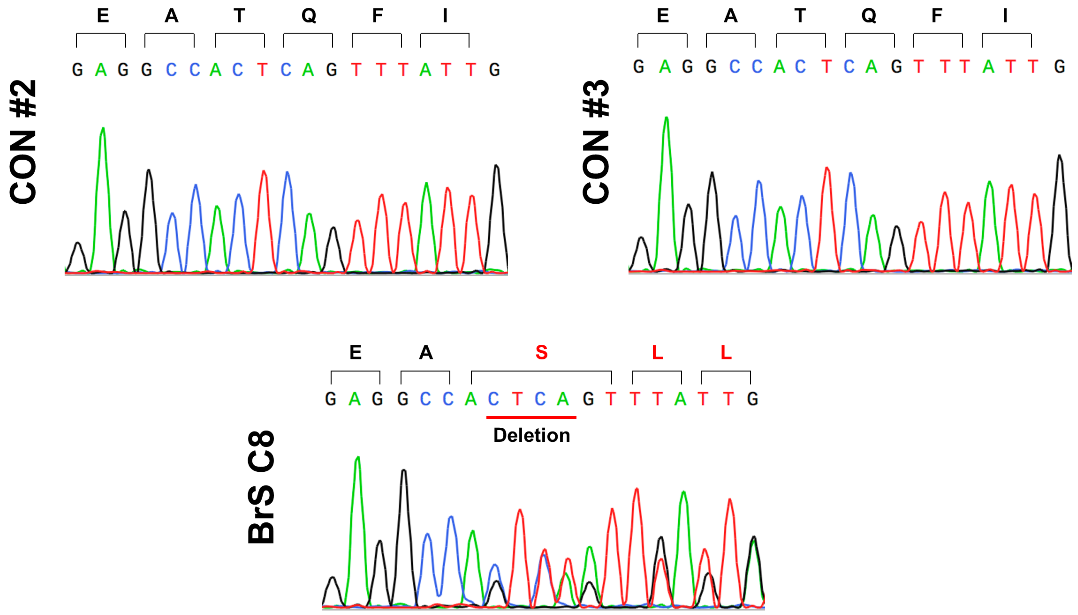

Supplemental Figure 3. DNA sequence chromatograms of the iPSC lines. Confirmation of the existence of the SCN5A T1788fs mutation in BrS iPSCs (BrS C8 iPSC line) but not in control iPSCs (CON#2 and CON#3 iPSC lines).

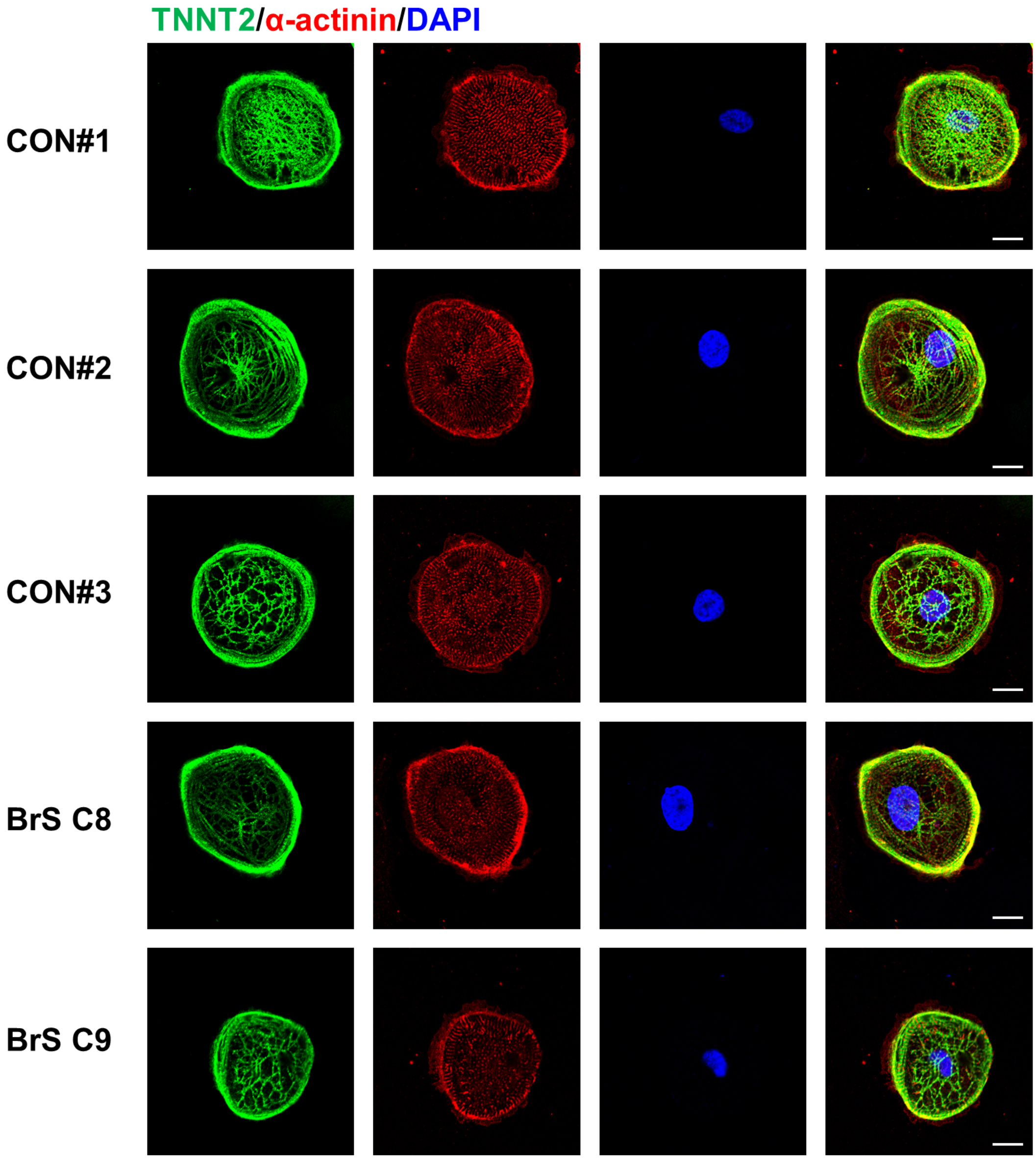

Supplemental Figure 4. Immunofluorescent staining of cardiac-specific markers in iPSC-CMs. Representative graphs of cardiac-specific staining by TNNT2 (green) and α-actinin (red) in control and BrS iPSC-CMs. DAPI indicates nuclear staining (blue). Scale bar, 20 μm.

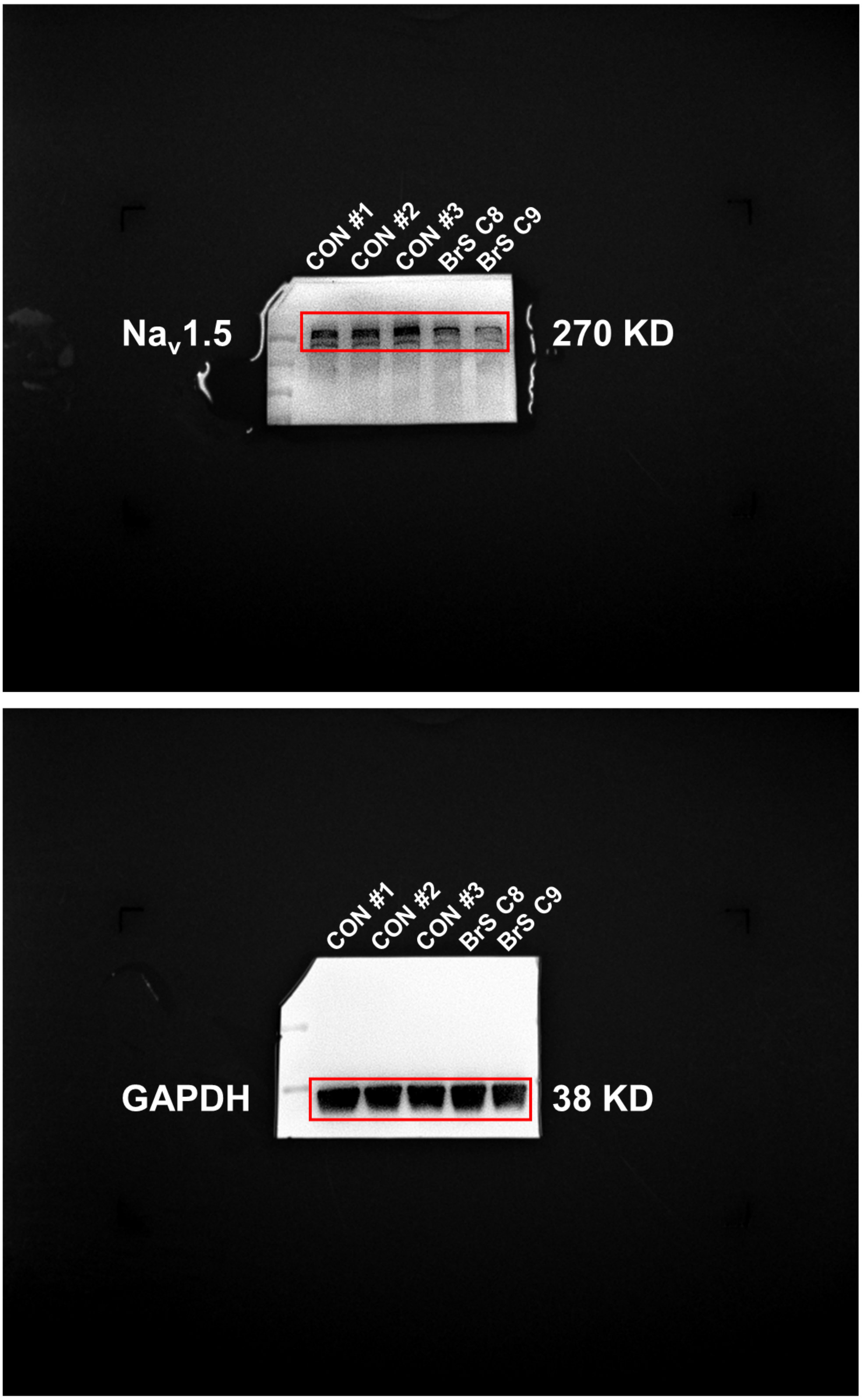

Supplemental Figure 5. Full length blots of Na$_V$1.5 expression in control and BrS iPSC-CMs. Red boxes indicate the cropped blots shown in Figure 3B.

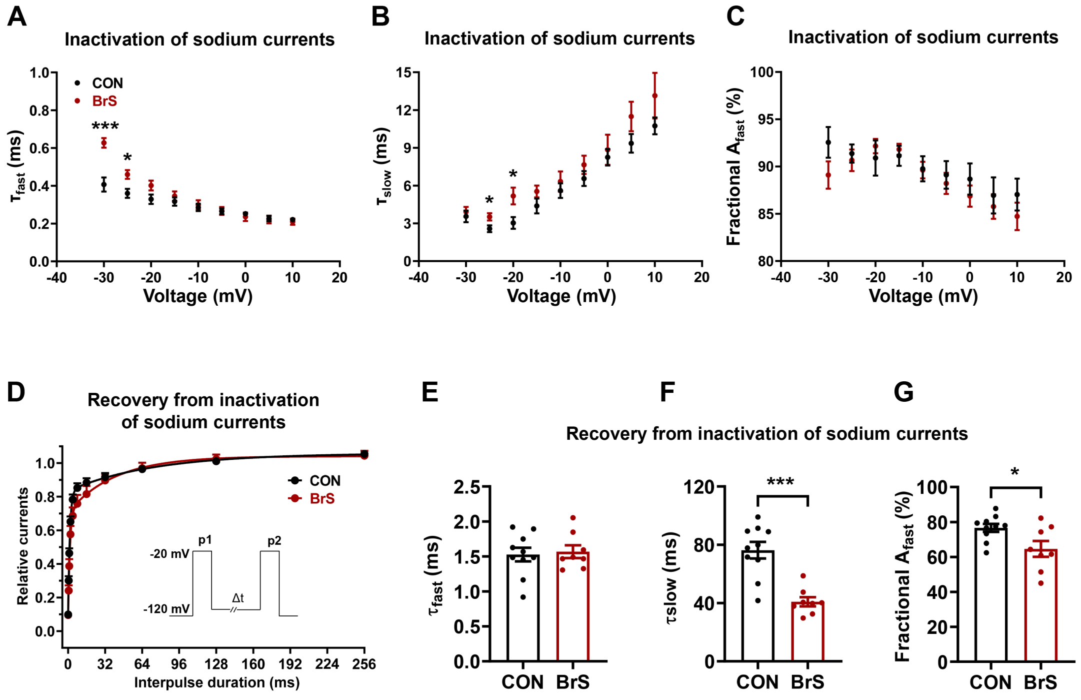

Supplemental Figure 6. Analysis of gating kinetics of sodium currents in control and BrS iPSC-CMs. A-C. Bar graphs to compare τ_{fast}, τ_{slow}, and fractional A_{fast} ($A_{fast}/A_{fast} + A_{slow}$) of inactivation of sodium currents at different voltages (from -30 mV to +10 mV with 5 mV increments) between control and BrS iPSC-CMs. n= 7-9 cells per group. $^{*}p< 0.05$ and $^{***}p< 0.001$. **D.** Summary of normalized recovery from inactivation of sodium currents fitted with a double-exponential function from control and BrS iPSC-CMs. **E-G.** Bar graphs to compare τ_{fast}, τ_{slow}, and fractional A_{fast} of recovery from inactivation of sodium currents between control and BrS iPSC-CMs. n= 8-10 cells per group. $^{*}p< 0.05$ and $^{***}p< 0.001$.

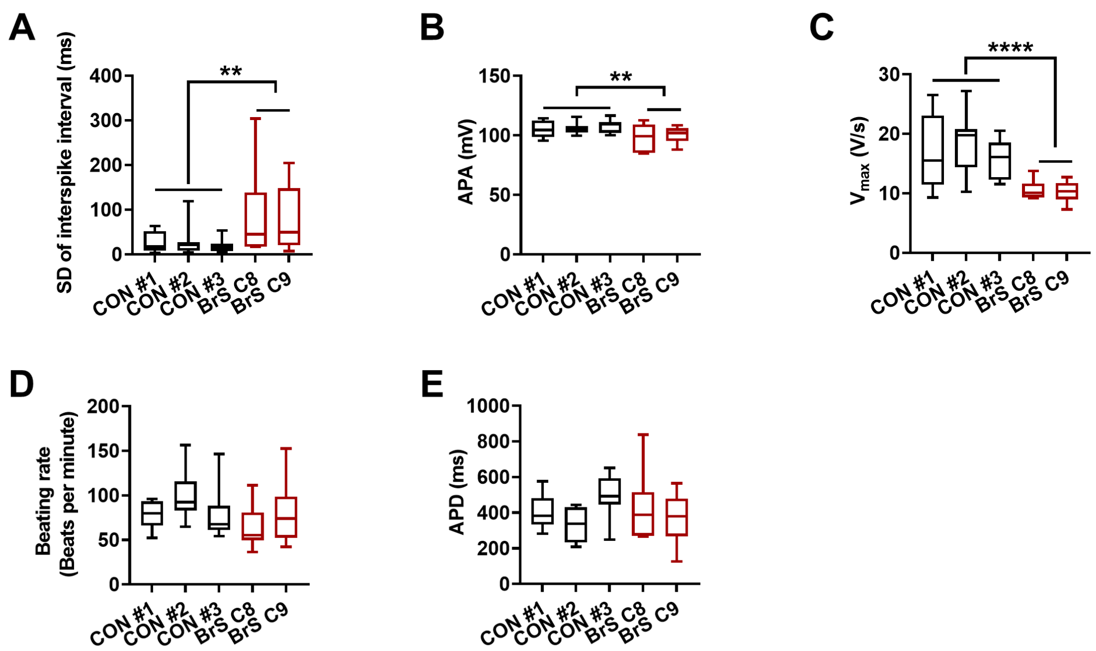

Supplemental Figure 7. Comparison of action potential parameters between control and BrS iPSC-CMs. A-E. Bar graphs to compare key parameters of action potentials between control and BrS iPSC-CMs, standard deviation (SD) of interspike intervals, action potential amplitude (APA), maximal upstroke velocity (V_{max}), beating rate and action potential duration (APD). n= 7-14 cells per group. $^{**}p< 0.01$ and $^{****}p< 0.0001$.

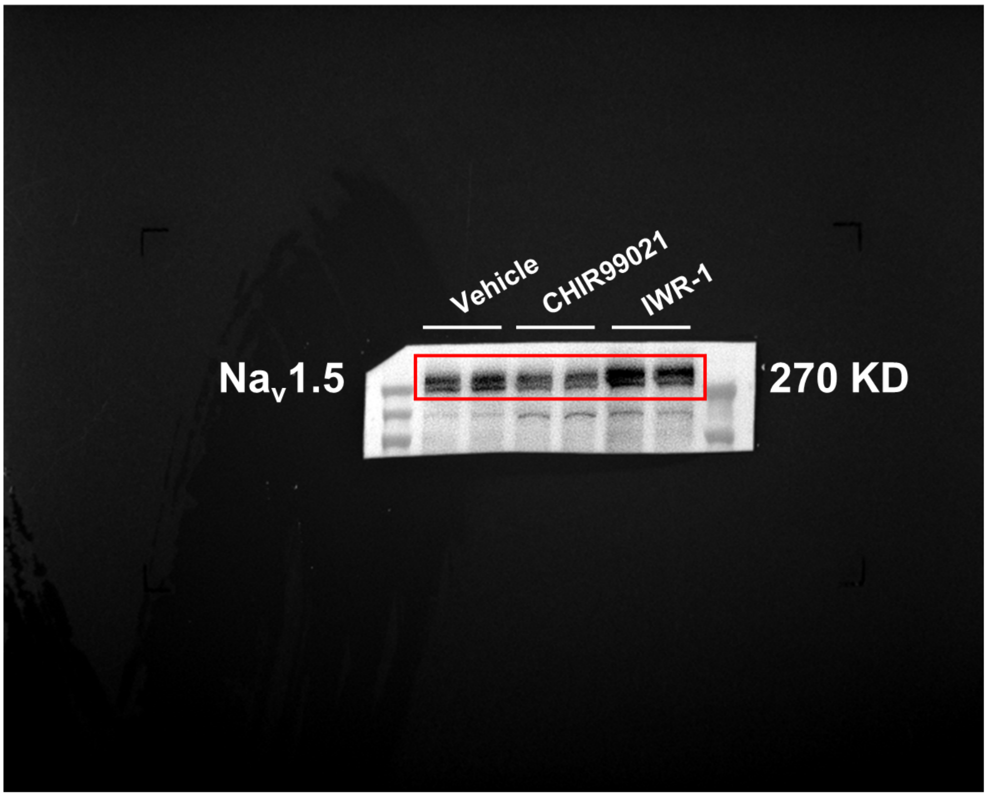

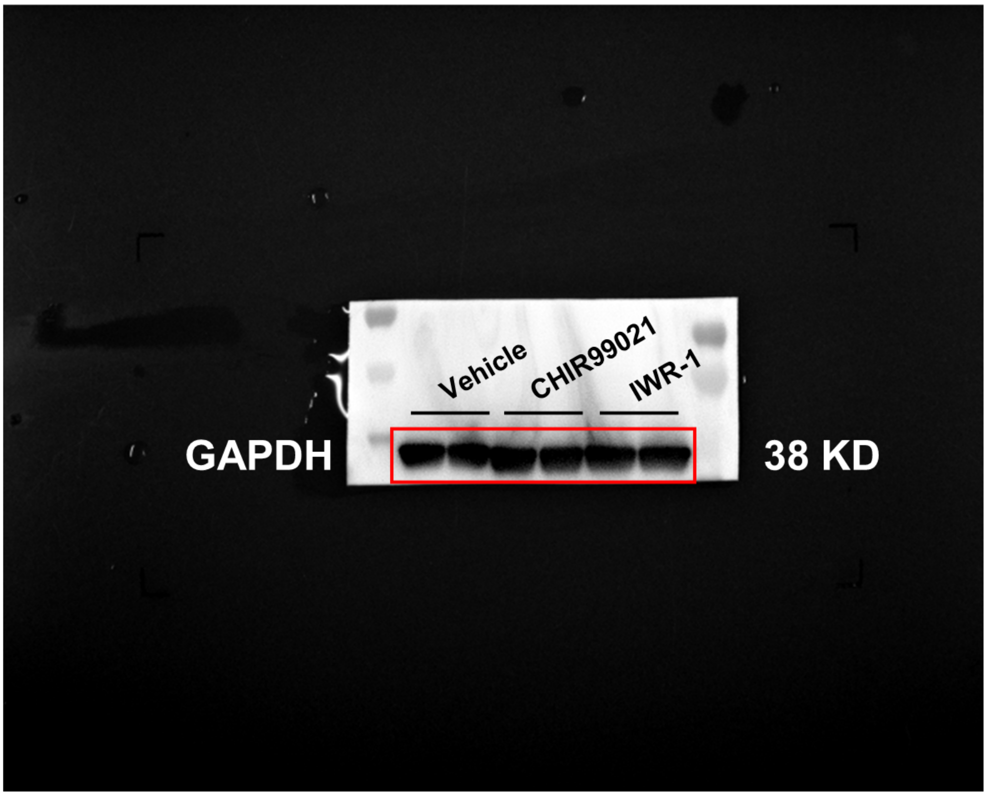

Supplemental Figure 8. Full length blots of $Na_V1.5$ expression in control iPSC-CMs treated with DMSO only (vehicle), 5 μM CHIR99021 or 10 μM IWR-1 for 72 hours, respectively. Red boxes indicate the cropped blots shown in Figure 4B.

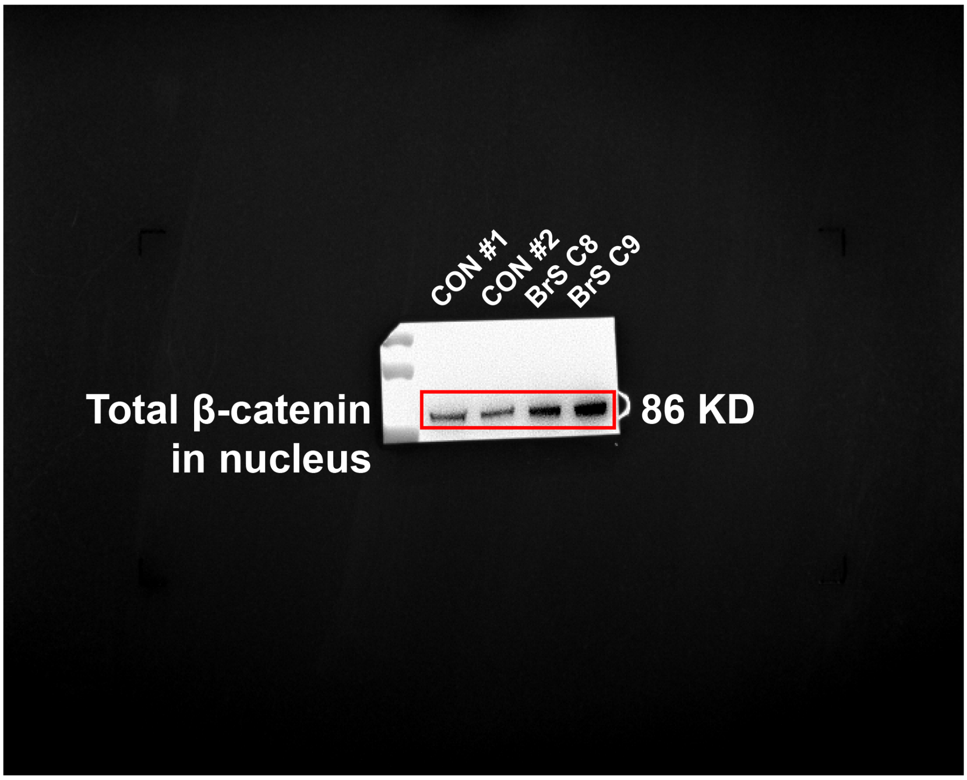

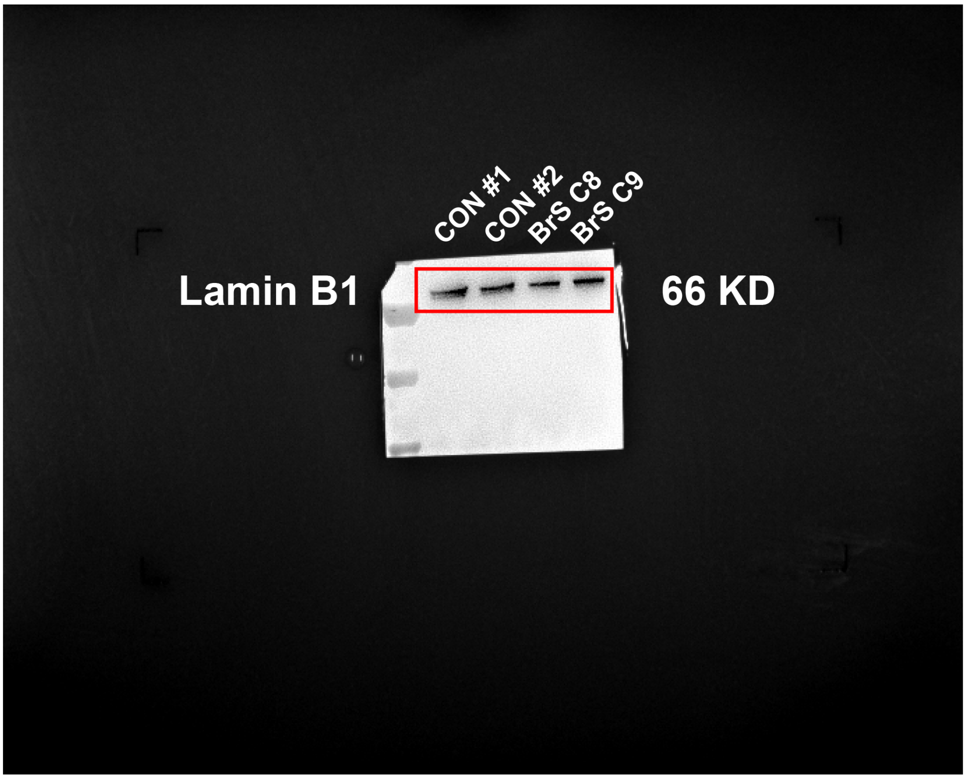

Supplemental Figure 9. Full length blots of nuclear expression of total β-catenin in control and BrS iPSC-CMs. Red boxes indicate the cropped blots shown in Figure 5B.

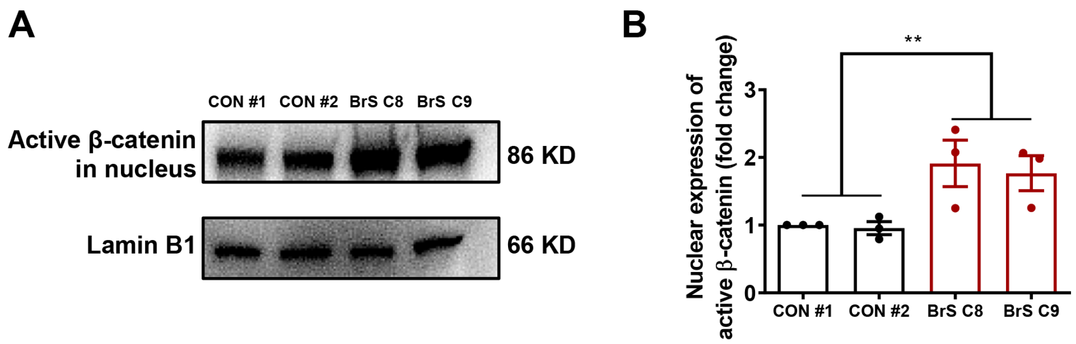

Supplemental Figure 10. Aberrant activation of Wnt/β-catenin in BrS iPSC-CMs. A. Western blot analysis of the nuclear expression of active β-catenin in control and BrS iPSC-CMs. Lamin B1 is used for the loading control. Full-length blots are presented in Supplemental Figure 11. **B.** Bar graph to compare the nucleus expression of active β-catenin in control and BrS iPSC-CMs. n= 3 culture replicates. $^{**}p < 0.01$.

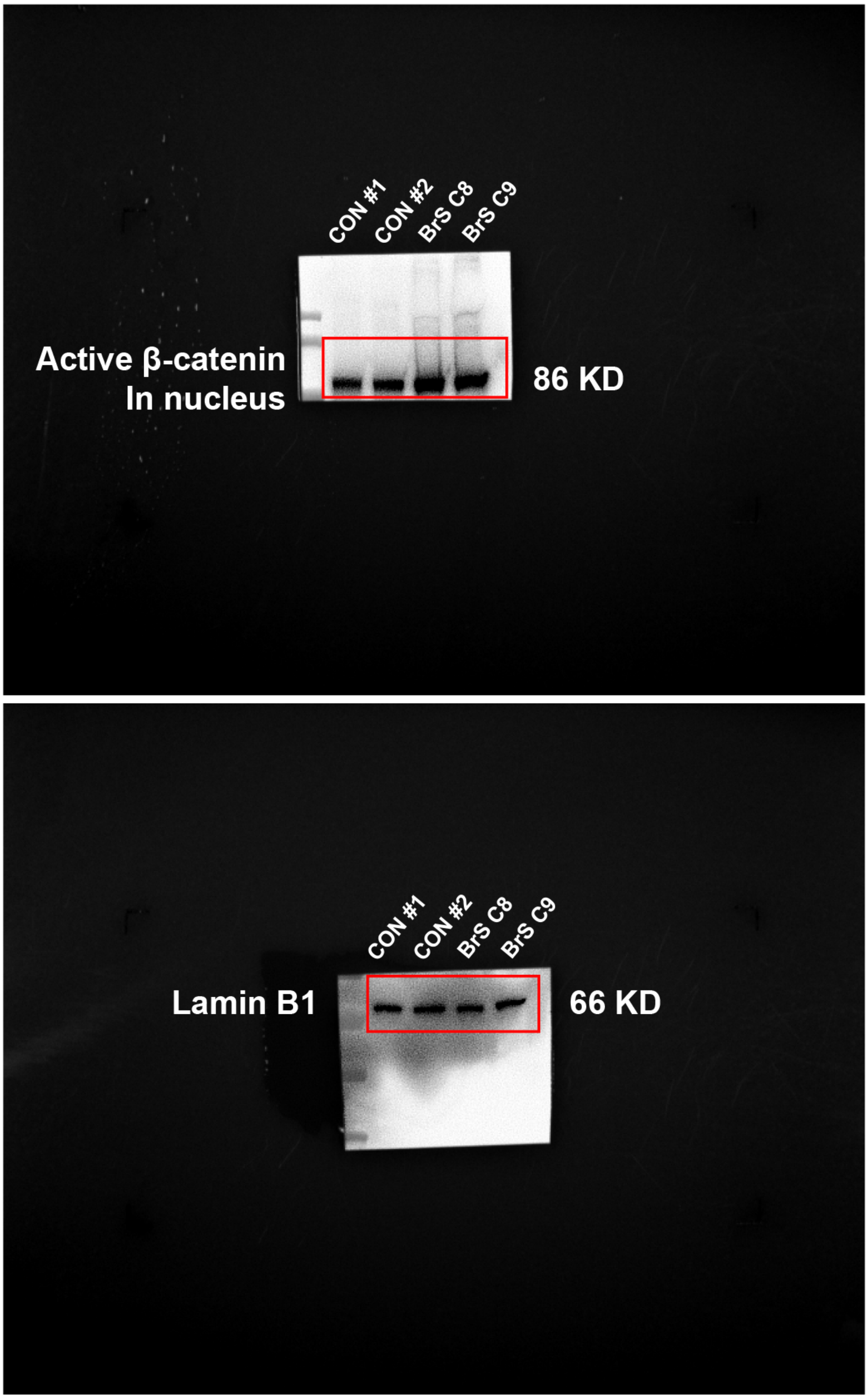

Supplemental Figure 11. Full length blots of nuclear expression of active β-catenin in control and BrS iPSC-CMs. Red boxes indicate the cropped blots shown in Supplemental Figure 10.

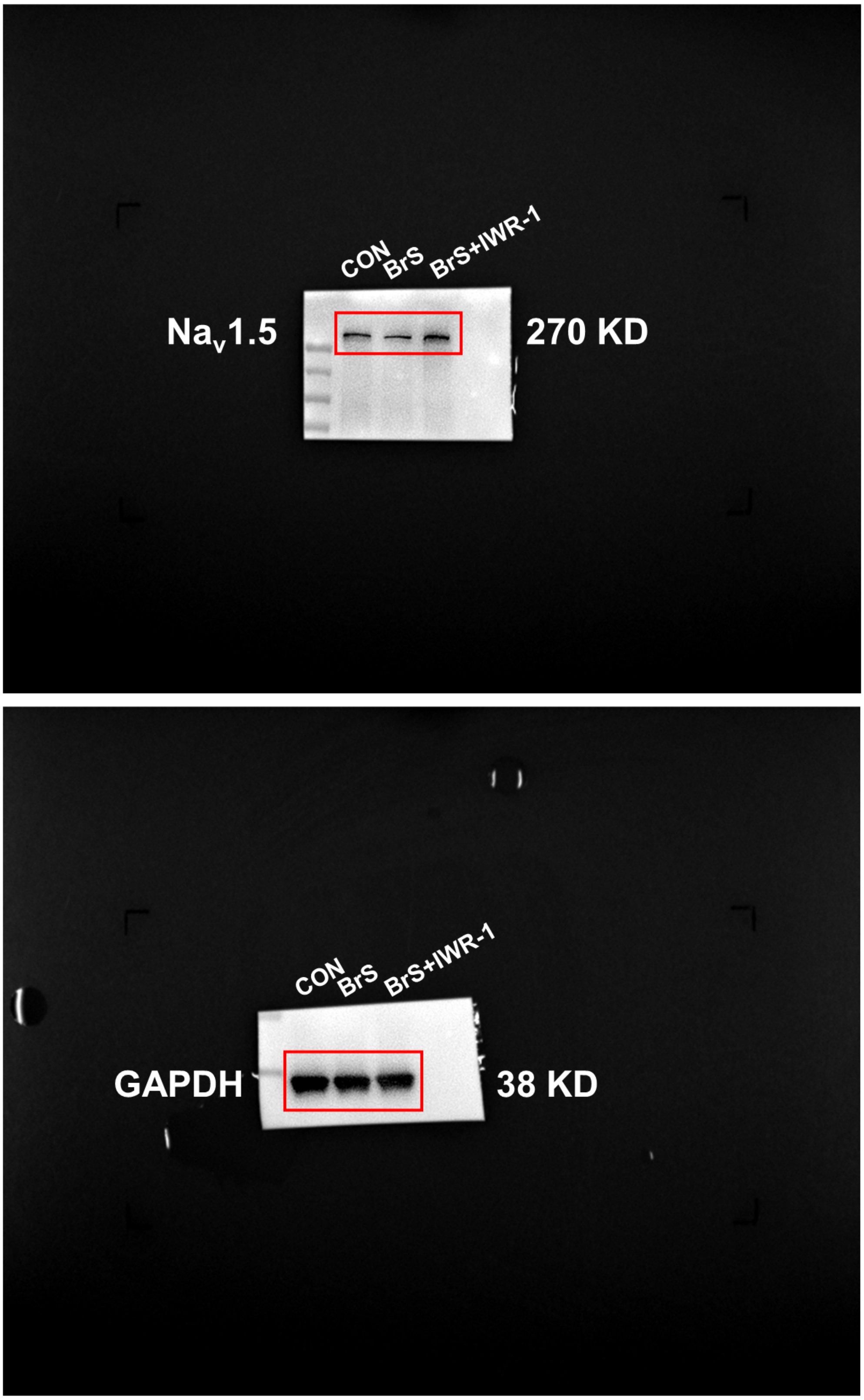

Supplemental Figure 12. Full length blots of $Na_V1.5$ expression in control iPSC-CMs, BrS iPSC-CMs treated with DMSO only, and BrS iPSC-CMs treated with 10 µM IWR-1. Red boxes indicate the cropped blots shown in Figure 6B.

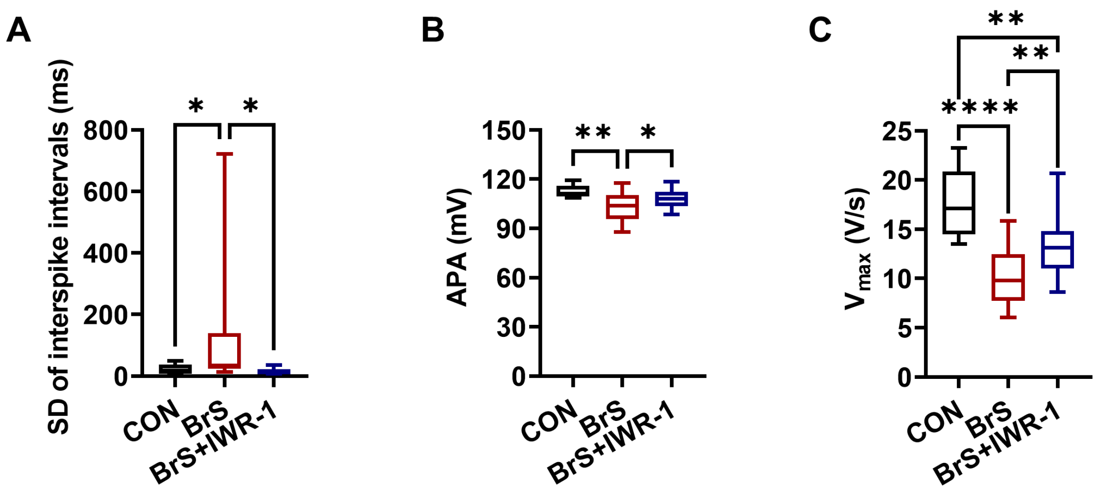

Supplemental Figure 13. Treatment of IWR-1 in BrS iPSC-CMs normalizes the action potential parameters. A-E. Bar graphs to compare SD of interspike intervals, APA and V_{max} between control iPSC-CMs, BrS iPSC-CMs treated with DMSO, and BrS iPSC-CMs treated with IWR-1. n= 9-23 cells per group. $^{*}p < 0.05$, $^{**}p < 0.01$ and $^{****}p < 0.0001$.

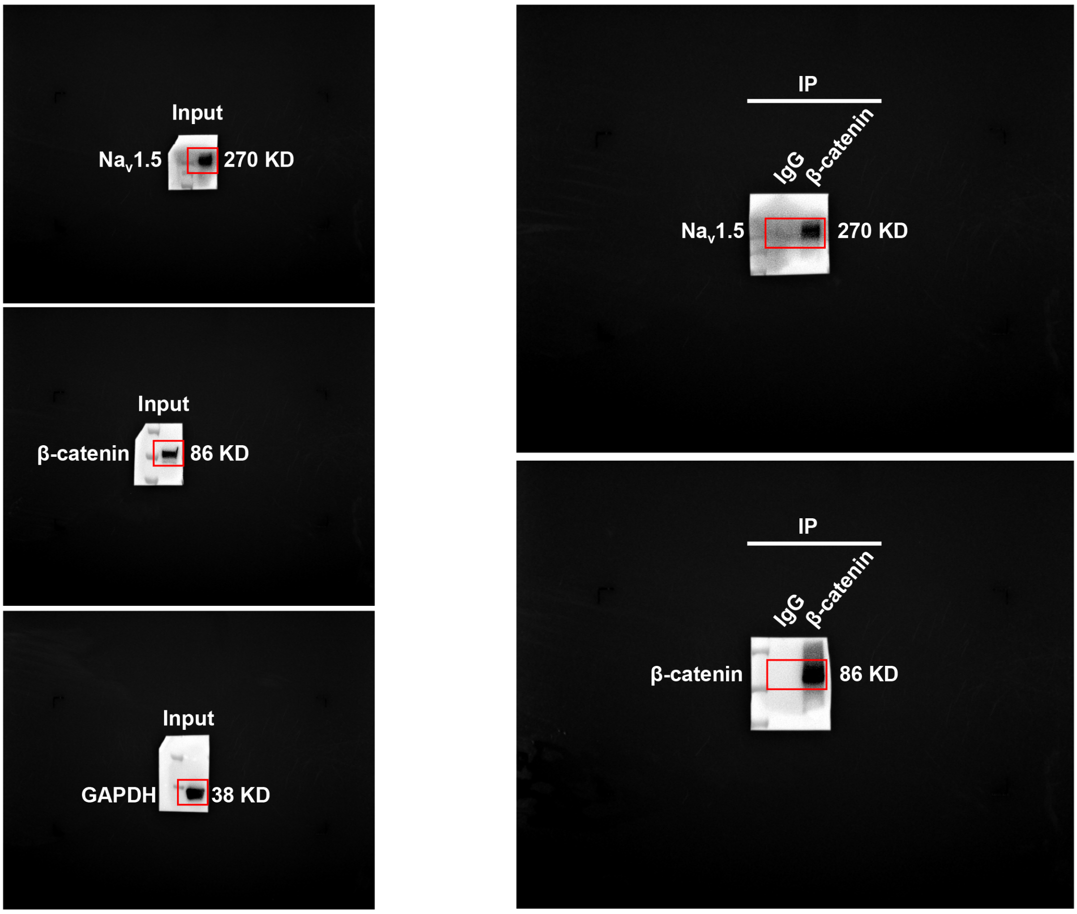

Supplemental Figure 14. Full length blots of the co-immunoprecipitation assay using antibody against β-catenin. Red boxes indicate the cropped blots shown in Figure 7A.

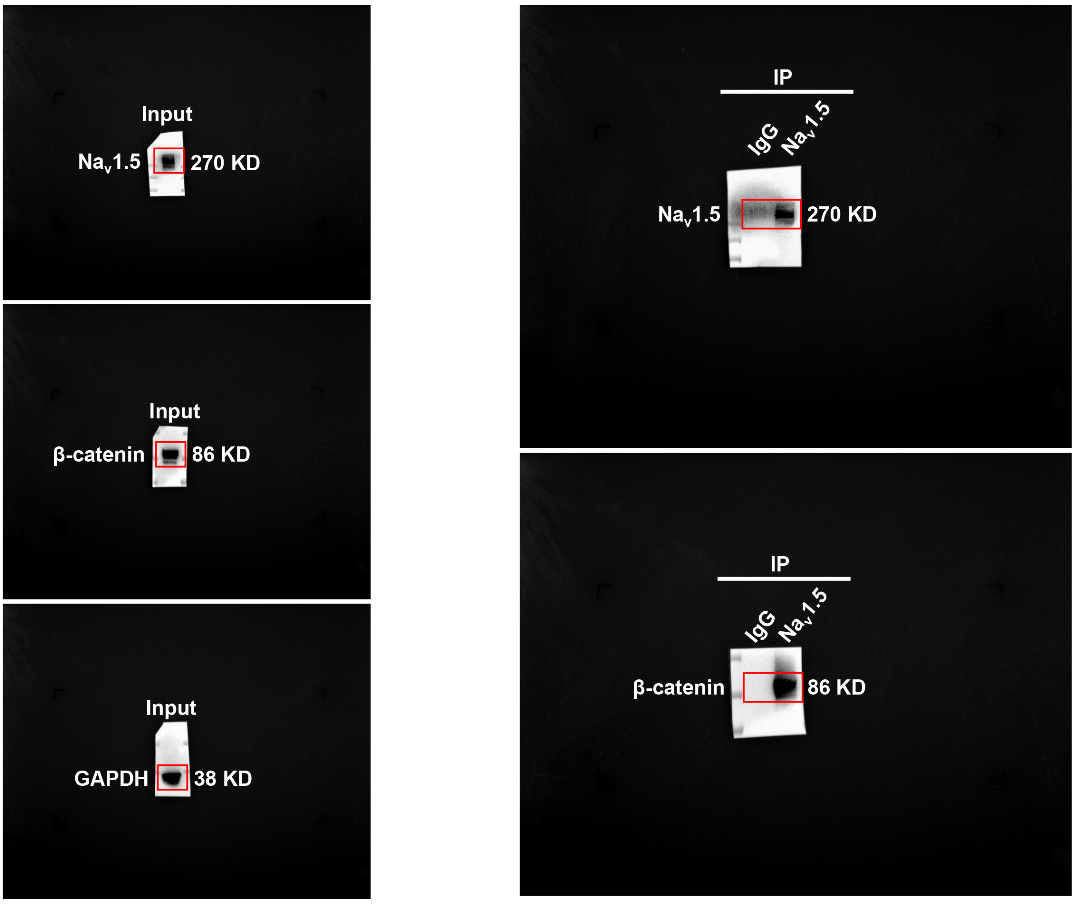

Supplemental Figure 15. Full length blots of the co-immunoprecipitation assay using antibody against Na_V1.5. Red boxes indicate the cropped blots shown in Figure 7B.

Supplemental Table 1. Primers used for qPCR in this study

Genes	Primer sequences		Gene Description
SCN5A	Forward	TCACCGCCATTTACACCTTTG	Sodium voltage-gated channel alpha subunit 5
	Reverse	GGTCCCGAAGGAAAGTGAACG	
GAPDH	Forward	GGTCGGAGTCAACGGATTTG	Glyceraldehyde-3-phosphate dehydrogenase
	Reverse	CGGTGCCATGGAATTTGCC	

Supplemental Table 2. Summary of recruited healthy control subjects and BrS patient in this study

	Age	Gender	Ethnicity	References
**Control #1**	26	Male	Han Chinese	No history of heart disease. ECG was normal.
**Control #2**	52	Male	Han Chinese	No history of heart disease. ECG was normal.
**Control #3**	32	Female	Han Chinese	No history of heart disease. ECG was normal.
**BrS patient**	28	Male	Han Chinese	Palpitations, amaurosis, and recurrent syncope. ICD recorded episodes of ventricular tachycardia and fibrillation. ECG showed a classical type 1 Brugada pattern.
